# Taxonomic Identification of Two Novel Genera and Four Novel Species of Lipolytic Floral-Associated Yeasts

**DOI:** 10.3390/jof12070521

**Published:** 2026-07-15

**Authors:** You-Jun Liao, Zi-Xuan Liu, Ya-Jing Yu, Ai-Hua Li

**Affiliations:** 1 China General Microbiological Culture Collection Center, Institute of Microbiology, Chinese Academy of Sciences, Beijing 100101, China; 2026003004@stu.njau.edu.cn (Y.-J.L.);; 2 College of Resources and Environmental Sciences, Nanjing Agricultural University, Nanjing 210095, China; 3 State Key Laboratory of Microbial Diversity and Innovative Utilization, Institute of Microbiology, Chinese Academy of Sciences, Beijing 100101, China

**Keywords:** floral yeast, novel species, lipolytic activity, *Fanglaniella*, *Polychromogenomyces*

## Abstract

Floral yeasts are generally recognized to exhibit high metabolic activities. We isolated 437 yeast strains from 45 flower samples collected from different plants in the Beijing Olympic Forest Park, a unique urban ecosystem harboring diverse plants, which facilitated a thorough survey of floral yeast diversity. Based on a sequence analysis of the D1/D2 domain of the large subunit ribosomal RNA gene (LSU rRNA) and the internal transcribed spacer region (ITS), these strains were assigned to 69 species, with *Filobasidium magnum*, *Starmerella bombicola*, *Teunia globosa*, *Aureobasidium pini*, and *Kwoniella ovata* being the dominant taxa. Ten representative strains were characterized comprehensively and identified as two novel genera and four novel species. Integrating the molecular data, genome information, and phenotypic/physiological traits, the ten novel yeast strains were described as six novel yeast taxa, including two novel species of two novel genera, *Fanglaniella lipolytica* gen. nov. sp. nov., *Polychromogenomyces tardus* gen. nov. sp. nov., and four novel species as *Pseudotremella jasmini* sp. nov., *Teunia pruni* sp. nov., *Kurtzmanomyces yulaniae* sp. nov., and *Trigonosporomyces otomorphus* sp. nov. Furthermore, qualitative profiling of the lipolytic activity across all six novel taxa was conducted using Tween 20 and Tween 80 plate culture assays, and the lipolytic functional genes were subsequently predicted. Our findings highlighted the high diversity of flower-inhabitant yeast strains and the potential lipolytic activity of floral yeasts.

## 1. Introduction

Lipolytic enzymes are hydrolytic biocatalysts with extensive applications across diverse industrial fields [1,2]. They primarily catalyze the hydrolysis of ester bonds in long-chain acylglycerols at the oil–water interface. Lipolytic activity can be widely produced by plants, animals, and microorganisms [3]. Notably, microbial lipolytic enzymes are more suitable for industrial use than their plant and animal counterparts, owing to their high yield, easy genetic manipulation, versatile catalytic properties, and year-round stable supply unaffected by seasonal fluctuations [4].

Fungi secrete lipolytic enzymes to utilize lipid substrates in their natural habitats. As ubiquitous enzymes, lipolytic enzymes exhibit distinct substrate specificity and strong stability across diverse physicochemical conditions, making them highly valuable for industrial exploitation [5]. Furthermore, many fungal lipolytic enzymes are secreted extracellularly, which reduces production costs and renders fungi superior to bacteria as industrial enzyme sources. Lipolytic enzymes from ascomycetous yeasts, including *Candida rugosa*, *Candida albicans*, *Candida viswanathii*, *Yarrowia deformans* or *Yarrowia lipolytica*, and *Pseudozyma antarctica* have been extensively investigated [6,7,8,9,10].

Currently, microbial diversity associated with floral nectar has become a popular research area, due to its great potential in biocontrol and pollination enhancement [11]. Floral microbiomes harbor a rich diversity of fungi and bacteria, which exert key functions in plant–insect interactions [12,13,14]. Both culture-dependent and culture-independent studies have revealed that the floral microbial community is dominated by a small number of yeast and bacterial genera. Among nectar-specialized yeasts, *Metschnikowia* is the most prevalent genus, followed by *Starmerella* and *Wickerhamiella*, all belonging to the phylum *Ascomycota*. Specifically, *Metschnikowia reukaufii* is recognized as the most ubiquitous nectar specialist across global ecosystems. Additionally, the generalist genera *Aureobasidium* (*Ascomycota*) and *Cryptococcus* (*Basidiomycota*) are also widely present in nectar, yet generally occur at lower abundances [15]. As nectar is an aqueous secretion containing high sugars, as well as amino acids, vitamins, lipids, and alkaloids [16,17], flowers are considered to be suitable biological substrates to isolate osmotolerant yeasts or lipolytic yeasts [17].

Herein, we isolated yeasts from floral samples collected throughout the Beijing Olympic Forest Park. This work aimed to characterize the diversity of flower-colonizing yeasts distributed in the park and to screen novel strains with valuable bioactive properties. The recovered isolates will expand the germplasm resources of floral-resident yeasts and provide promising candidates for the exploration of novel biocatalytic enzymes.

## 2. Materials and Methods

### 2.1. Sampling and Isolation of Yeast Strains

Flower samples were collected from the Beijing Olympic Forest Park, China (40.0177° N, 116.3861° E) in April 2019 and April 2025. In total, 45 samples representing 21 flower species were acquired, including *Yulania denudata*, *Yulania liliiflora*, *Jasminum nudiflorum*, *Prunus persica ‘Albo-plena’*, *Prunus persica ‘Kikumomo’*, and *Syringa oblata*, as well as other flower varieties. All samples were collected with sterile tools, placed into sterile plastic bags, kept at 4 °C, and transported to the laboratory for immediate processing.

Approximately 5 g of petal or stamen samples were suspended in 100 mL of sterile 0.5% NaCl solution, vigorously shaken for 10 min, followed by soaking for 3 h. This suspension was serially diluted to a concentration of 10^−5^, and 200 µL aliquots were plated onto yeast extract–malt extract (YM) agar plates (1% yeast extract, 2% malt extract, 0.4% glucose, and 2% agar) and potato dextrose agar (PDA, 0.4% potato extract, 2% glucose, and 2% agar) plates. Each medium was supplemented with 100 μg/mL of ampicillin and 100 μg/mL of streptomycin sulfate. The plates were incubated at 25 °C until visible colonies formed. Different yeast morphotypes were purified by re-streaking on PDA plates. The purified yeast strains were preserved at the China General Microbiological Culture Collection Center (CGMCC), Beijing, China, using lyophilization (freeze-drying) and cryopreservation in 10% glycerol at −80 °C.

### 2.2. Molecular Phylogenetic Analysis

Genomic DNA was extracted using a commercial DNA Extraction Kit (AirLab BioDev). The D1/D2 domain of the large subunit (LSU) rRNA gene was amplified by PCR with the primers NL1/NL4, following the protocol of Kurtzman and Robnett [18]. PCR was performed with an initial predenaturation at 94 °C for 1 min, followed by 35 amplification cycles consisting of denaturation at 94 °C for 1 min, annealing at 52 °C for 45 s, and elongation at 72 °C for 45 s, with a final extension step at 72 °C for 5 min. The internal transcribed spacer (ITS) region was amplified using the primers ITS1 and ITS4 as described by White et al. [19]. The PCR profile included an initial predenaturation at 95 °C for 5 min, followed by 35 amplification cycles consisting of denaturation at 95 °C for 30 s, annealing at 55 °C for 45 s, and elongation at 72 °C for 45 s, with a final extension step at 72 °C for 5 min [20]. All acquired sequences were subjected to BLAST searches against the GenBank database (https://blast.ncbi.nlm.nih.gov; accessed on 5 February 2026). Reference sequences of related type strains were retrieved from GenBank, aligned in MEGA 7.0 [21], and manually edited as needed. Given intragenomic polymorphism within the fungal ITS region [22], phylogenetic analyses of the novel strains were performed using the concatenated D1/D2 domain and ITS region, or the combined D1/D2 and ITS sequence datasets. Phylogenetic trees were constructed using the maximum likelihood (ML) method [23] in MEGA 7.0. Prior to the ML phylogenetic tree reconstruction, the best-fit evolution model was determined via jModelTest using the Bayesian Information Criterion (BIC) [24]. Branch support was assessed by bootstrap analysis with 1000 replicates [25]. Only bootstrap values greater than 50% were displayed on the phylogenetic trees [26].

### 2.3. Whole Genome Sequencing and Annotation

Genome sequencing was performed on the Illumina NovaSeq X plus platform (Inc., San Diego, CA, USA) at Majorbio Science and Technology Ltd. (Beijing, China), employing a paired-end sequencing approach. Raw reads were subjected to quality control prior to de novo assembly with SOAPdenovo v2.0 (BGI Hong Kong Research Institute, Hong Kong, China; http://soap.genomics.org.cn/soapdenovo.html, 14 May 2026). The completeness of the draft genome assemblies was assessed using CEGMA (UC Davis Genome Center, Davis, CA, USA; http://korflab.ucdavis.edu/datasets/cegma/, 14 May 2026). The genomic DNA quality was verified via agarose gel electrophoresis and quantified using a Nanophotometer NP80 Mobile (Implen GmbH, Munich, Germany). Qualified DNA samples were sent to Majorbio Bio Ltd. (Beijing, China) for whole-genome sequencing. DNA fragmentation was conducted using the Covaris M220 system (Covaris, Inc., Woburn, MA, USA), and paired-end libraries were sequenced on the Illumina NovaSeq X Plus platform. Raw reads were filtered with the NGS QC Toolkit v2.3 (National Institute of Plant Genome Research, New Delhi, India; http://www.nipgr.res.in/ngsqctoolkit.html, 14 May 2026) to remove vector and adapter sequences. Quality control criteria were defined as follows: Phred quality score ≥ 20, and over 80% of each read retained as high-quality bases. Clean reads were then assembled de novo using SOAPdenovo v2.0 [27]. The completeness of the draft genome assemblies was evaluated using both BUSCO v4 (University of Geneva & Swiss Institute of Bioinformatics, Geneva, Switzerland; https://busco.ezlab.org/, 14 May 2026) and CEGMA pipelines (UC Davis Genome Center, Davis, CA, USA; http://korflab.ucdavis.edu/datasets/cegma/, 14 May 2026) [28].

Genome annotation was conducted by homology searches against the Virulence-related genes were identified by sequence alignment against the Database of Fungal Virulence Factors (DFVF, University of Nebraska, Lincoln, NE, USA; http://sysbio.unl.edu/DFVF/, 14 May 2026) using DIAMOND v2.0 (Max Planck Institute for Developmental Biology, Tübingen, Germany; https://github.com/bbuchfink/diamond, 14 May 2026). Antibiotic resistance genes (ARGs) were predicted from the Comprehensive Antibiotic Resistance Database (CARDv1.1.3, McMaster University, Hamilton, ON, Canada; http://arpcard.Mcmaster.ca, 14 May 2026). Secreted proteins were predicted using SignalP (Technical University of Denmark, Lyngby, Denmark; http://www.cbs.dtu.dk/services/SignalP, 14 May 2026). Transporter proteins were annotated via the Transporter Classification Database (TCDB; University of California, San Diego, CA, USA; http://www.tcdb.org/, 14 May 2026). Transmembrane protein-coding genes were identified using TMHMM (Technical University of Denmark, Lyngby, Denmark; http://www.cbs.dtu.dk/services/TMHMM/, 14 May 2026).

### 2.4. Phenotypical Characterization

Morphological, biochemical, and physiological characterizations were carried out following the standard methods described by Kurtzman et al. [29]. Carbon assimilation was tested in a liquid medium of yeast nitrogen base (Difco, 291940), and nitrogen assimilation assays were performed using a liquid yeast carbon base (Difco, 239110), with starved inocula employed for the latter [30]. Carbohydrate fermentation was determined using Durham tubes at 25 °C [31]. To observe pseudohyphae formation, pre-cultured strains were cultivated on both corn meal agar (CMA, 2.5% corn starch, and 2% agar) and PDA at 25 °C for 2 weeks, employing a cover glass to create an oxygen-limited environment [32]. Temperature tolerance was assessed by culturing strains on YM agar. Cell morphology was examined via light microscopy and scanning electron microscopy (SEM, SU8180, Hitachi, Tokyo, Japan) after incubation on PDA at 25 °C for 3 days.

Sexual structure induction was conducted by culturing individually and in combination on YM agar, PDA, and CMA at 25 °C for up to 2 months with periodic observation [33]. Ballistoconidium production of all novel species was tested via the inverted-plate method on CMA at 25 °C [34]. After incubating for 3 to 14 days, slides were prepared and observed under microscope to detect any discharged spores [35].

### 2.5. Extracellular Enzyme Activity Assays

Extracellular enzyme activities were determined after the strains were cultivated for 2 weeks of growth at 25 °C, following the enzymatic assay procedures described below. (1) Amylolytic activity: strains were grown on YM agar containing 0.2% soluble starch. Plates were overlaid with 1 mL of iodine solution. A clear halo around colonies against a purple background indicated positive enzymatic activity [36]; (2) Cellulase activity: Cultivation was performed on YM agar supplemented with 0.5% carboxymethylcellulose [37]. Plates were flooded with 1 mg/mL Congo red solution and left for 15 min before the dye was poured off. The plates were then soaked in 1 M NaCl solution for another 15 min. A clear halo on the red-stained agar confirmed cellulolytic activity [38]; (3) Protease activity: Protease activity was assessed on YM agar supplemented with 2% casein. After incubation of the strains, a positive reaction was indicated by a clear zone surrounding the colony in the opaque medium [39]; (4) Chitinase activity: Strains were cultured on YM agar with 2.5% purified chitin. Chitinase activity was identified by the formation of a clear halo zone surrounding colonies [40]; (5) Lipolytic activity: Strains were cultured on medium composed of 1% Bacto Peptone, 0.5% NaCl, 0.01% CaCl_2_•2H_2_O, 2% agar, and 1% Tween 20. Lipolytic activity was evidenced by a white precipitate encircling colonies [36]; (6) Xylanase activity: Isolates were grown on YM agar containing 0.5% xylan. A clear halo around the colonies indicated positive xylanase activity [41]; (7) Pectinase activity: Strains were cultured on 0.67% YNB medium supplemented with 1% pectin. After following plates with 1% hexadecyltrimethylammonium bromide solution, a clear zone against a red background confirmed pectinase activity [40]; (8) Esterase activity: Strains were cultured on a medium containing 1% Bacto Peptone, 0.5% NaCl, 0.01% CaCl_2_•2H_2_O, 2% agar, and 1% Tween 80 [42]. Esterase activity was determined by the appearance of a white precipitate encircling colonies.

## 3. Results

### 3.1. Diversity of Isolated Floral Yeast Strains

A total of 437 yeast strains were isolated from 45 collected flower samples and assigned to 69 yeast species, including 12 ascomycetous and 57 basidiomycetous species. The dominant genera were *Starmerella*, *Teunia*, *Kwoniella*, *Aureobasidium*, *Vishinacozyma*, *Cystobasidium*, *Dioszegia*, and *Cytofilobasidium*.

**Figure 1:**
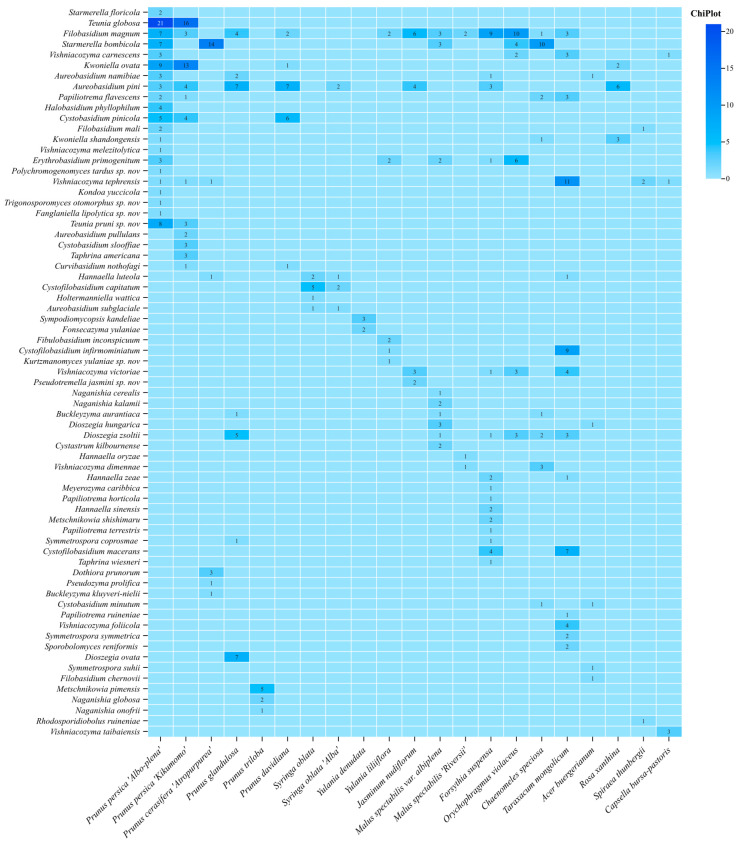
The yeast strains isolated from flowers of different plants.

The isolation counts of the yeast species recovered from various ornamental flower hosts are shown in Figure 1, which revealed striking divergence in yeast community diversity among the floral hosts. The dichotomy between host-endemic unique yeasts and cross-host shared yeasts was clearly revealed.

*Prunus persica* ‘*Albo-plena*’ harbored the highest yeast diversity and quantity, with a total of 21 taxa and a maximum of 21 isolates of a single yeast taxon, followed by *Taraxacum mongolicum* with a total of 15 yeast taxa. Yeast communities isolated from the flowers of different *Prunus persica* cultivars exhibited distinct differences. This indicates that closely related ornamental flowers do not possess more similar yeast community structures, and no significant correlation was detected between plant phylogenetic relatedness and floral yeast assemblages. A large suite of yeast taxa were host-specific, only isolated from a single floral species. The high-abundance signature of unique yeasts exclusive to *Prunus persica ‘Albo-plena’* include *Starmerella floricola* and *Teunia globosa. Kwoniella ovata* was uniquely enriched on *Prunus persica ‘Kikumomo’*. Host-specific rare yeasts also covered *Kondoa yuccicola*, *Cystofilobasidium capitatum*, *Sporobolomyces renuinensis*, and *Rhodosporidiobolus taibaiensis*, among others, each restricted to individual hosts. By contrast, only a small number of generalist shared yeasts colonize multiple flower hosts at low isolate counts (1–5 strains), including *Aureobasidium namibiae*, *Aureobasidium pini*, *Papiliotrema flavescens*, *Hannaella luteola*, *Meyerozyma caribbica*, *Symmetrospora coprosmae*, and *Filobasidium chernovii*, among others. No yeast taxon was recovered from all plant hosts. Overall, each floral yeast community was dominated by host-specific endemic species, while shared generalist yeasts exist merely as minor accessory members, indicating the strong host selectivity of floral yeasts.

**Figure 2:**
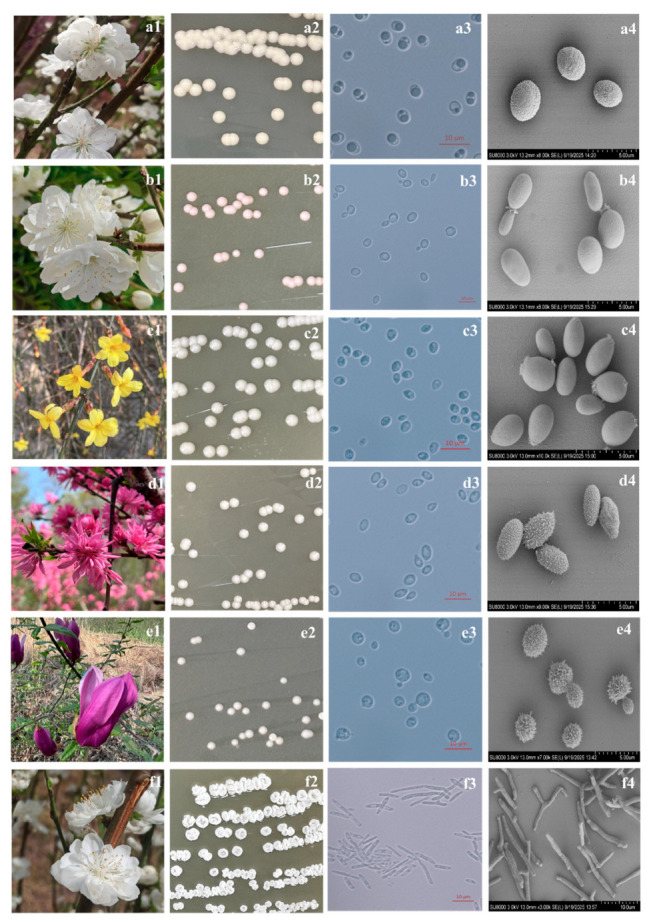
The isolation sources (**a1**,**b1**,**c1**,**d1**,**e1**,**f1**), colonies on PDA after 7–10 days at 25 °C (**a2**,**b2**,**c2**,**d2**,**e2**,**f2**), cell morphology observed by light microscope (**a3**,**b3**,**c3**,**d3**,**e3**,**f3**) and SEM (**a4**,**b4**,**c4**,**d4**,**e4**,**f4**) after 3 days of growth on PDA at 25 °C. (**a1**–**a4**) *Fanglaniella lipolytica* CGMCC 2.6218; (**b1**–**b4**) *Polychromogenomyces tardus* CGMCC 2.8784; (**c1**–**c4**) *Pseudotremella jasmini* CGMCC 2.6068; (**d1**–**d4**) *Teunia pruni* CGMCC 2.8783; (**e1**–**e4**) *Kurtzmanomyces yulaniae* CGMCC 2.8812; (**f1**–**f4**) *Trigonosporomyces otomorphus* CGMCC 2.6214. Scale bars: 10 μm (**a3**,**b3**,**c3**,**d3**,**e3**,**f3**,**f4**); 5 μm (**a4**,**b4**,**c4**,**d4**,**e4**). (**a1**,**b1**,**f1**): flowers of *Prunus persica ‘Albo-plena’*; (**c1**): flowers of *Jasminum nudiflorum*; (**d1**): flowers of *Prunus persica ‘Kikumomo’*; (**e1**): flowers of *Yulania liliiflora*.

Based on the ITS and D1/D2 LSU rDNA sequence comparisons, ten isolates were preliminarily assigned to six putative novel species; phylogenetic reconstruction and polyphasic phenotypic characterizations further validated their novel taxonomic status. Comprehensive data covering the isolation source and the colonial and cellular morphological characteristics of these novel strains are compiled in Figure 2.

### 3.2. Phylogenetic Analyses of the Novel Yeast Taxa

As the ten novel isolates exhibited significant sequence differences in both the D1/D2 domain and the ITS regions from all known species, they were proposed as six novel species of two novel genera and four known genera.

**Figure 3:**
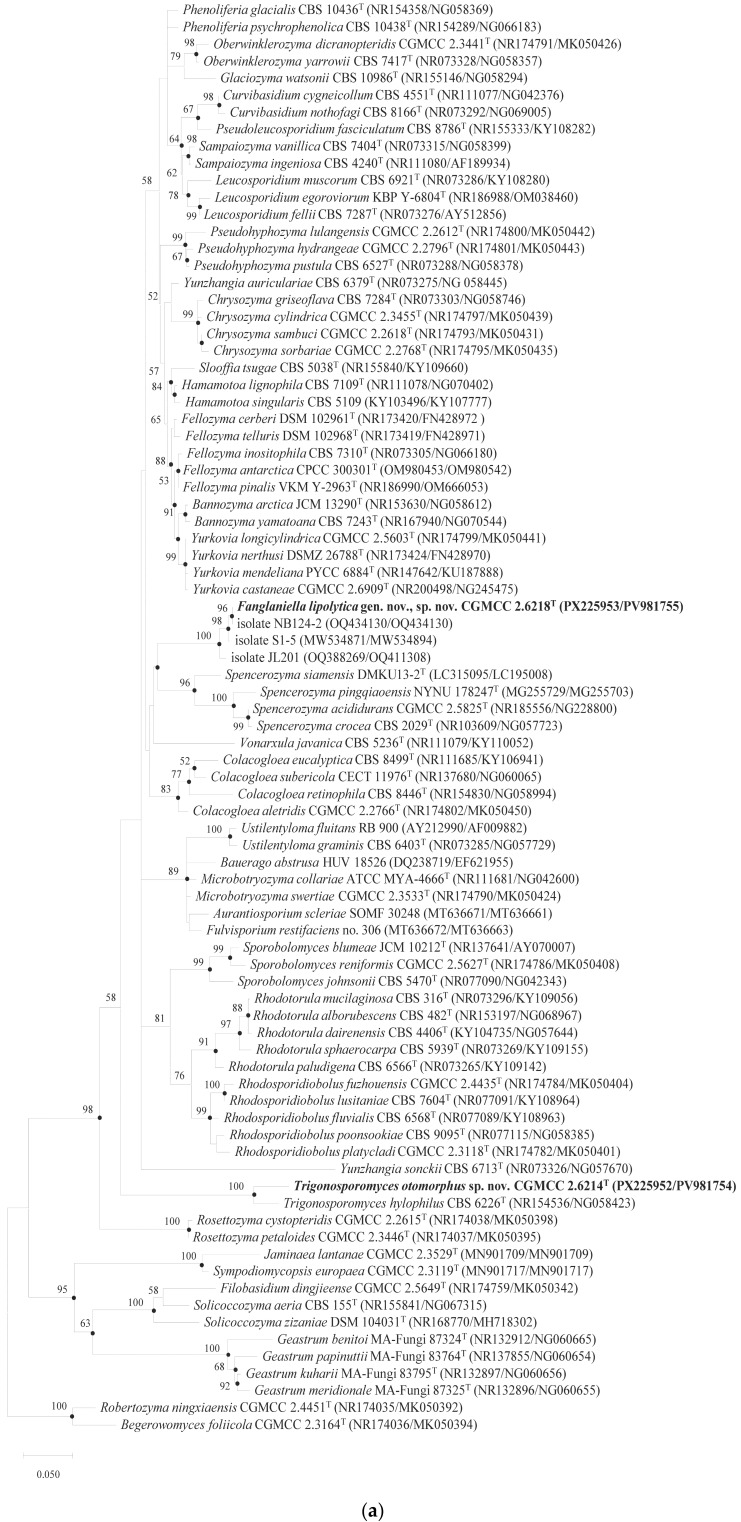

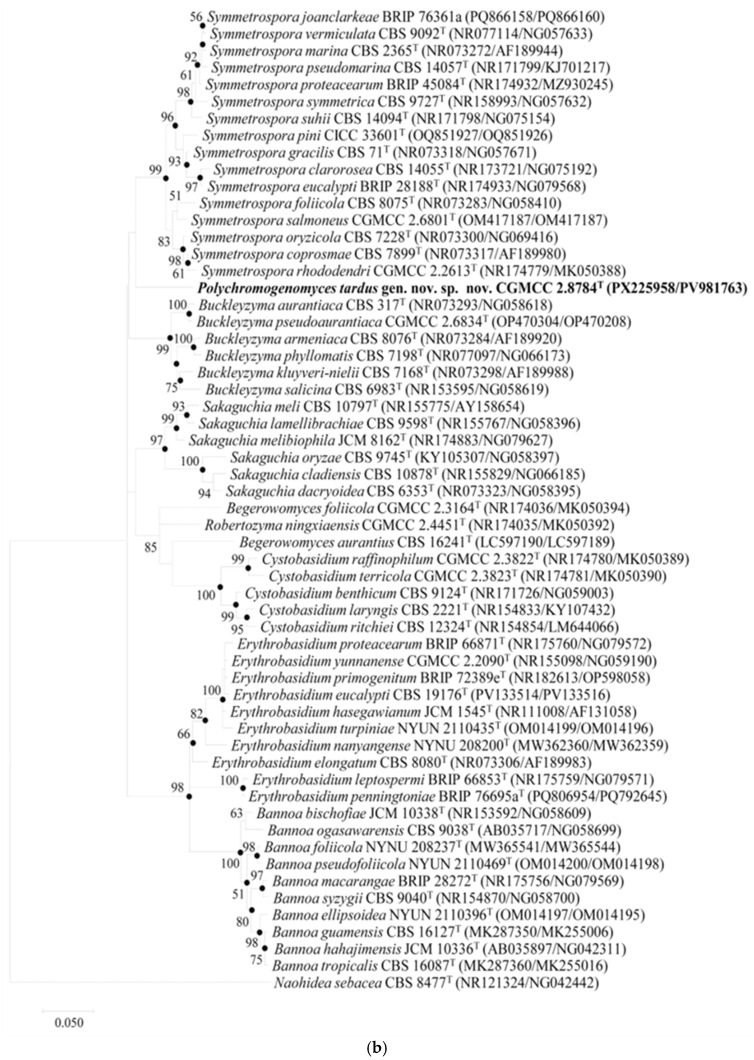

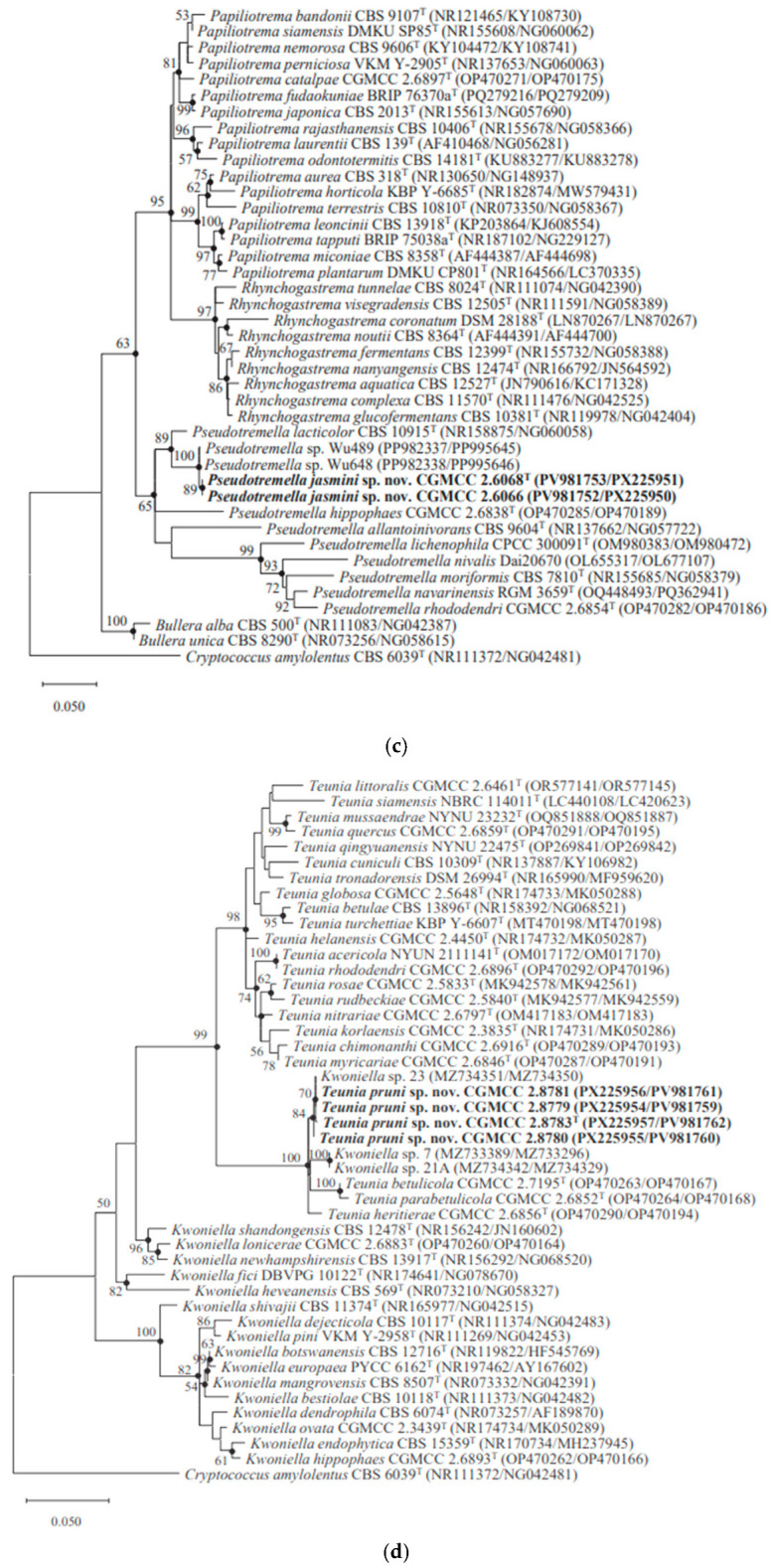

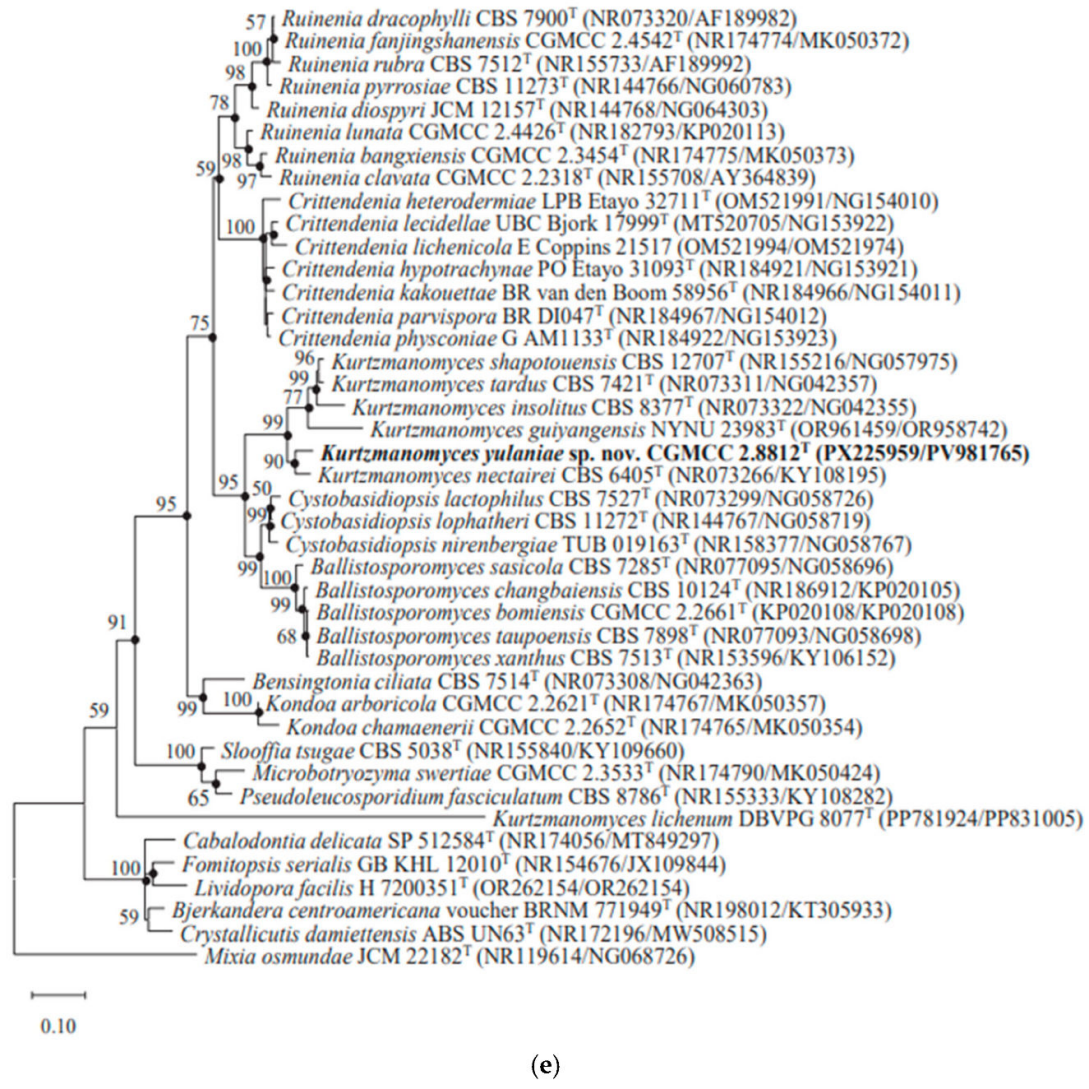
Maximum likelihood phylogenetic tree based on the concatenated sequences of the D1/D2 domain of LSU rRNA and ITS region showing the relationships of these novel strains with their related species. (**a**) *Fanglaniella lipolytica* CGMCC 2.6218 and *Trigonosporomyces otomorphus* CGMCC 2.6214. *Robertozyma ningxiaensis* CGMCC 2.4451^T^ and *Begerowomyces foliicola* CGMCC 2.3164^T^ were used as the outgroup. Scale bar represents 0.05 substitutions per nucleotide position. (**b**) *Polychromogenomyces tardus* CGMCC 2.8784. *Naohidea sebacea* CBS 8477^T^ was used as the outgroup. Scale bar represents 0.05 substitutions per nucleotide position. (**c**) *Pseudotremella jasmini* CGMCC 2.6068. *Cryptococcus amylolentus* CBS 6039^T^ was used as the outgroup. Scale bar represents 0.05 substitutions per nucleotide position. (**d**) *Teunia pruni* CGMCC 2.8783. *Cryptococcus amylolentus* CBS 6039^T^ was used as the outgroup. Scale bar represents 0.05 substitutions per nucleotide position. (**e**) *Kurtzmanomyces yulaniae* CGMCC 2.8812. *Mixia osmundae* JCM 22182^T^ was used as the outgroup. Scale bar represents 0.1 substitutions per nucleotide position. Bootstrap values from 1000 replications are shown at branch points.

The strain CGMCC 2.6218 (original code S2A6) was isolated from the flowers of *Prunus persica ‘Albo-plena’*. Pairwise D1/D2 LSU sequence comparisons revealed identities ranging from 93.60% to 94.46% against the closely related type strains *Yurkovia longicylindrica* CGMCC 2.5603^T^ (94.46%), *Pseudohyphozyma lulangensis* CGMCC 2.2612^T^ (94.36%), *Glaciozyma watsonii* CBS 10986^T^ (94.28%), *Chrysozyma griseoflava* CBS 7284^T^ (93.68%), and *Chrysozyma sambuci* CGMCC 2.2618^T^ (93.60%). The ITS-based alignments yielded even lower sequence similarities (86–88%), with representative species of *Yamadamyces*, *Pseudohyphozyma*, and *Fellozyma*. Despite the disparate similarity values, the BLASTN results consistently affiliated this strain within the class *Microbotryomycetes*. Combined low interspecific sequence identity and weak phylogenetic nodal support justified the establishment of a novel genus to accommodate CGMCC 2.6218, for which the name *Fanglaniella lipolytica* gen. nov. sp. nov. is designated. Multiple unpublished NCBI-deposited strains, including NB124-2 (from sorghum roots), S1-5 (from a Xizang plant sample) and JL 221 (from the phyllosphere of *Cardamine hirsuta*), clustered robustly with CGMCC 2.6218 into a single monophyletic lineage, confirming their conspecific status (Figure 3a).

The strain CGMCC 2.8784 (original code BHBT-Y41) was isolated from *Prunus persica ‘Albo-plena’*. The D1/D2 LSU sequence comparison showed 98.45–98.77% identity between this strain and type strains of *Symmetrospora*, including *S. rhododendri* CGMCC 2.2613^T^, *S. salmoneus* CGMCC 2.6801^T^, *S. coprosmae* CBS 7899^T^, *S. foliicola* CBS 8075^T^, and *S. oryzicola* CBS 7228^T^. By contrast, the ITS BLASTN results yielded pairwise similarities below 90% against all valid *Symmetrospora* species. Notably, a consistent 30 bp deletion was identified in the LSU sequence of CGMCC 2.8784^T^ when aligned with its *Symmetrospora* relatives. Maximum likelihood phylogeny built on the concatenated ITS and D1/D2 LSU datasets placed CGMCC 2.8784 as a well-separated outgroup forming an independent monophyletic lineage outside the *Symmetrospora* clade (Figure 3b). Such molecular evidence firmly supported the establishment of a separate genus distinct from *Symmetrospora*, for which we propose the name *Polychromogenomyces tardus* gen. nov., sp. nov.

The strains CGMCC 2.6068 (original code YGRX8-1) and CGMCC 2.6066 (original code YGRX1-2), were recovered from different flower specimens of *Jasminum nudiflorum*. Their identical D1/D2 LSU and ITS sequences confirmed conspecificity. The sequence alignment results of the D1/D2 and ITS sequences indicated that the two strains exhibited the closest phylogenetic relationship with *Pseudotremella lacticolor* CBS 10915^T^. Specifically, they differed from it by 20 nucleotide discrepancies (3.53%, 17 substitutions and 3 indels) across the D1/D2 domain and 68 mismatches (11.67%, 50 substitutions and 18 indels) within the ITS region. In the ML-tree reconstructed based on the concatenated sequences of the D1/D2 domains of the LSU rRNA gene and the ITS region, the strains CGMCC 2.6068 and CGMCC 2.6066 grouped with unpublished isolates Wu 489 and Wu 648 into a robust monophyletic clade, which further clustered with *Pseudotremella lacticolor* CBS 10915ᵀ (Figure 3c). The result strongly supported that the two strains represent a novel species, for which the name *Pseudotremella jasmini* sp. nov. was proposed, with CGMCC 2.6068 designated as the holotype strain.

Four strains, including CGMCC 2.8779 (original code BHBT-P11), CGMCC 2.8780 (original code BHBT-P15), CGMCC 2.8781 (original code JHT-P15), and CGMCC 2.8783 (original code JHT-P17), were recovered from *Prunus persica ‘Albo-plena’* (CGMCC 2.8779 and CGMCC 2.8780) and *Prunus persica ‘Kikumomo’* (CGMCC 2.8781 and CGMCC 2.8783), respectively. These isolates shared identical D1/D2 LSU and ITS sequences or differed by merely one to two nucleotide positions, corroborating their conspecific status. Sequence comparisons of the D1/D2 and ITS regions revealed that they differed from the nearest relative *Teunia heritierae* CGMCC 2.6856^T^ by 7 nucleotide substitutions (1.25%) within the D1/D2 domain and 15 variable sites (2.65%, 13 substitutions and two indels) across the ITS region. Larger sequence divergence was detected against other congeneric taxa: 8 D1/D2 substitutions (1.57%) plus 33 ITS variations (5.66%, 30 substitutions and 3 indels) relative to *Teunia betulicola* CGMCC 2.7195^T^, and 9 D1/D2 substitutions (1.61%) plus 42 ITS polymorphisms (7.05%, 37 substitutions and 5 indels) versus *Teunia parabetulicola* CGMCC 2.6852^T^. In the ML phylogenetic tree reconstructed based on the concatenated D1/D2 LSU and ITS region, the four isolates first clustered with the unpublished strain *Kwoniella* sp. 23 and subsequently formed a well-supported monophyletic clade with their closest relatives in the genus *Teunia*, *Teunia heritierae* CGMCC 2.6856^T^, *Teunia betulicola* CGMCC 2.7195^T^, and *Teunia parabetulicola* CGMCC 2.6852^T^ (Figure 3d), plus two unpublished isolates *Kwoniella* sp. strain 7 and strain 21A. All aforementioned reference isolates were recovered from tree leaf samples collected in Xizang. Consistent with pairwise sequence divergence and phylogenetic evidence, the four strains constituted a novel species within *Teunia*. We therefore proposed the name *Teunia pruni* sp. nov., with CGMCC 2.8783 assigned as the holotype.

The strain CGMCC 2.8812 (original code YLH-Y2), isolated from *Yulania liliiflora*, was phylogenetically affiliated to the genus *Kurtzmanomyces.* In the concatenated D1/D2 LSU–ITS ML tree, it formed a well-supported subclade with *K. nectairei* CBS 6405^T^ before grouping consecutively with *K. insolitus* CBS 8377^T^, *K. shapotouensis* CBS 12707^T^, *K. tardus* CBS 7421^T^, and *K. guiyangensis* NYNU 23983^T^ (Figure 3e). Pairwise sequence comparisons against its nearest neighbor, *K. nectairei* CBS 6405^T^, revealed 19 nucleotide substitutions (3.37%) across the D1/D2 domain and 111 variable sites within the ITS region (17.12%, 93 substitutions plus 18 indels). Such substantial molecular divergence confirmed CGMCC 2.8812 as an undescribed member of *Kurtzmanomyces*, for which we propose the name *Kurtzmanomyces yulaniae* sp. nov.

The strain CGMCC 2.6214 (original code S6A3), isolated from the flowers of *Prunus persica ‘Albo-plena’*, fell into the *Trigonosporomyces* lineage and formed a tight subclade with *Tri. hylophilus* CBS 6226^T^ in the ML phylogenetic tree based on the concatenated D1/D2 LSU and ITS sequences (Figure 3a). A pairwise sequence comparison against this nearest relative revealed 32 nucleotide substitutions (5.34%) in the D1/D2 domain and 88 variable positions in the ITS region (10.95%, 67 substitutions and 21 indels). The considerable molecular divergence robustly supports the establishment of a novel species, *Trigonosporomyces otomorphus* sp. nov., accommodating strain CGMCC 2.6214.

### 3.3. Extracellular Enzyme Profiling

**Figure 4:**
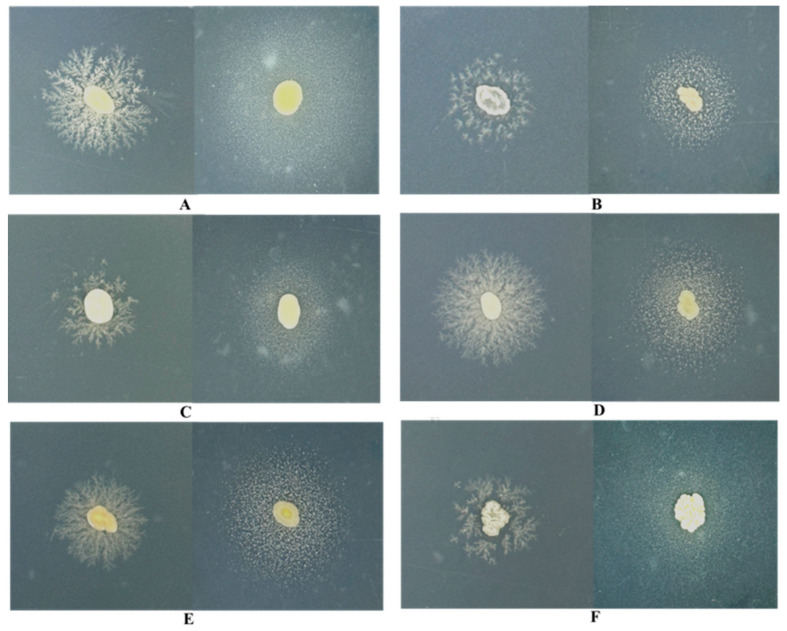
Qualitative results of the extracellular lipolytic activity assays of novel yeast isolates after two weeks of cultivation. For each group (**A**–**F**), the left showed the results on Tween 20 plates, and the right showed the results on Tween 80 plates. (**A**) *Fanglaniella lipolytica* CGMCC 2.6218; (**B**) *Polychromogenomyces tardus* CGMCC 2.8784; (**C**) *Pseudotremella jasmini* CGMCC 2.6068; (**D**) *Teunia pruni* CGMCC 2.8783; (**E**) *Kurtzmanomyces yulaniae* CGMCC 2.8812; (**F**) *Trigonosporomyces otomorphus* CGMCC 2.6214.

Eight extracellular enzyme assays (amylase, cellulase, protease, chitinase, lipolytic enzyme, xylanase, pectinase, and esterase) were performed across the six novel yeast species. All six isolates yielded positive hydrolysis against Tween 20 and Tween 80 substrates, confirming their capacity to synthesize extracellular lipolytic enzymes (Figure 4). None of the strains displayed detectable activity for amylase, cellulase, chitinase, xylanase, or pectinase. Furthermore, extracellular protease activity was detected in *Fanglaniella lipolytica* CGMCC 2.6218, *Trigonosporomyces otomorphus* CGMCC 2.6214, and *Pseudotremella jasmini* CGMCC 2.6068, though the activity was relatively weak (Figure S3).

### 3.4. Genome Sequence Analysis and Genome Features

The draft genomes of the type strains of six proposed novel species—*Fanglaniella lipolytica* CGMCC 2.6218ᵀ, *Polychromogenomyces tardus* CGMCC 2.8784ᵀ, *Pseudotremella jasmini* CGMCC 2.6068ᵀ, *Teunia pruni* CGMCC 2.8783ᵀ, *Kurtzmanomyces yulaniae* CGMCC 2.8812ᵀ, and *Trigonosporomyces otomorphus* CGMCC 2.6214ᵀ—were sequenced (Table 1), with sequencing coverage depths of 327×, 218×, 108×, 282×, 360×, and 454×, respectively.

*Fanglaniella lipolytica* CGMCC 2.6218ᵀ had a genome size of 17.6 Mb and a GC content of 55.5%. Its draft genome consisted of 324 contigs (>1000 bp) with a total length of 17,616,076 bp; the largest contig was 802,846 bp, and the contig N50 value was 165,752 bp.

*Polychromogenomyces tardus* CGMCC 2.8784ᵀ possessed a genome size of 23.5 Mb and a GC content of 54.5%. The draft genome comprised 322 contigs (>1000 bp) totaling 23,477,971 bp, with the largest contig of 852,679 bp and a contig N50 value of 205,933 bp.

*Pseudotremella jasmini* CGMCC 2.6068ᵀ had a genome size of 21.1 Mb and a GC content of 52%. Its draft genome included 63 contigs (>1000 bp) with a total length of 21,071,928 bp; the largest contig was 2,188,978 bp, and the contig N50 value was 856,280 bp.

*Teunia pruni* CGMCC 2.8783ᵀ had a genome size of 20.8 Mb and a GC content of 55.5%. The draft genome consisted of 156 contigs (>1000 bp) totaling 20,808,089 bp, with the largest contig of 1,515,199 bp and a contig N50 value of 446,252 bp.

*Kurtzmanomyces yulaniae* CGMCC 2.8812ᵀ possessed a genome size of 18.2 Mb and a GC content of 56.5%. Its draft genome included 165 contigs (>1000 bp) with a total length of 18,214,608 bp; the largest contig was 1,112,172 bp, and the contig N50 value was 462,428 bp.

*Trigonosporomyces otomorphus* CGMCC 2.6214ᵀ had a genome size of 12.1 Mb and a GC content of 52.5%. The draft genome comprised 111 contigs (>1000 bp) totaling 12,110,825 bp, with the largest contig of 1,381,140 bp and a contig N50 value of 298,846 bp.

A genomic analysis of the ITS region revealed that strains *Pseudotremella jasmini* CGMCC 2.6068ᵀ, *Trigonosporomyces otomorphus* CGMCC 2.6214ᵀ, and *Teunia pruni* CGMCC 2.8783ᵀ each possessed only one copy of the ITS region, indicating an absence of intragenomic polymorphism that would compromise species delimitation in the present study. The numbers of predicted protein-coding genes in *Fanglaniella lipolytica* CGMCC 2.6218ᵀ, *Polychromogenomyces tardus* CGMCC 2.8784ᵀ, *Pseudotremella jasmini* CGMCC 2.6068ᵀ, *Teunia pruni* CGMCC 2.8783ᵀ, *Kurtzmanomyces yulaniae* CGMCC 2.8812ᵀ, and *Trigonosporomyces otomorphus* CGMCC 2.6214ᵀ were 4931, 6495, 7371, 6415, 7842, and 4553, respectively. The corresponding numbers of predicted tRNA genes were 76, 71, 62, 64, 15, and 99, respectively.

### 3.5. Genome Annotation and Predicted Genes Responsible for Lipolytic Enzymes

**Figure 5:**
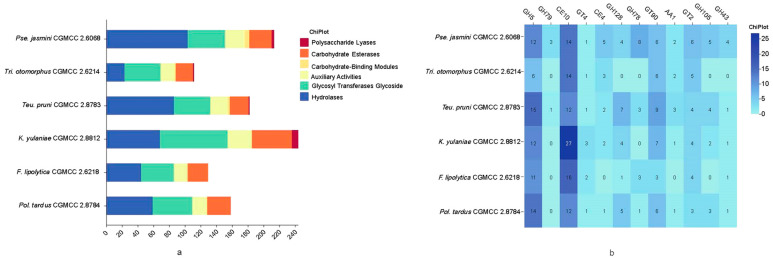
CAZyme gene family profiles: (**a**) Stacked bar plot of CAZyme category distribution; (**b**) Heatmap of carbohydrate-active enzyme (CAZyme) families including glycoside hydrolase (GH), glycosyltransferase (GT), carbohydrate esterase (CE) and auxiliary activity (AA) identified in the six novel yeast strains.

Genome annotation was conducted for all six novel strains, followed by systematic profiling of carbohydrate-active enzymes (CAZymes). As illustrated in Figure 5a, their CAZyme repertoires were dominated by abundant glycoside hydrolase (GH) genes, a conserved trait commonly observed across conventional yeasts. In particular, *Teunia pruni* CGMCC 2.8783^T^, *Pseudotremella jasmini* CGMCC 2.6068^T^, and *Polychromogenomyces tardus* CGMCC 2.8784^T^ possessed the highest GH copy numbers, implying strong genetic potential for polysaccharide degradation via glycosidic bond cleavage (Figure 5a). Among the identified GH families, GH5 was the most abundant clade, whose core function was the hydrolysis of *β*-glucan. Another prominent characteristic specific to these novel strains was the widespread distribution of CE10 family members in their genomes (Figure 5b). CE10 enzymes were characterized by broad-spectrum lipolytic activity, and they mediated key physiological processes including carbon source utilization, lipid metabolism, and xenobiotic detoxification via a serine hydrolase catalytic mechanism, thereby acting as pivotal enzymes that enable yeast to adapt to heterogeneous environments. Notably, previously reported yeast strains harboring CE10 family members, such as *Yarrowia lipolytica* YALI0E15568p and *Candida tropicalis* CEL1, typically exhibited robust lipolytic activities. This finding, to a certain extent, elucidated the underlying mechanism responsible for the lipolytic activity of these novel yeast strains.

In order to predict which genes potentially contributed to the lipolytic activity of these strains, we systematically analyzed the responsible genes based on the Swiss-Prot and NR databases. As the results indicated, beyond the CE10 family genes, we additionally identified other candidate genes potentially responsible for the lipolytic activities of these strains through systematic screening against the Swiss-Prot and NR databases. As shown in Table 2, all six strains harbored TGL genes encoding triacylglycerol lipase, which mediated the hydrolysis of long-chain fatty acid esters. Moreover, five of these strains, with the exception of *Teunia pruni* CGMCC 2.8783, carried genes encoding hormone-sensitive lipase (HSL) (Table 2). These HSLs exhibited broad-spectrum lipolytic activity and represented one of the key genes responsible for the degradation of Tween 20/80 by the strains. In addition, *Kurtzmanomyces yulaniae* CGMCC 2.8812 harbored the gene protein C1039.03 (SPAC1039.03), which was an uncharacterized *α/β* hydrolase superfamily protein in *Schizosaccharomyces pombe*, putatively involved in hydrolytic reactions, such as ester/amidase activity and potentially regulated by nutrient or environmental stress. The virulence factor and the antibiotic resistance gene presented in the genome of these novel yeast strains are listed in Tables S3 and S4.

Notably, the identification of these homologous genes merely provided bioinformatic predictions, and we have clearly stated that additional functional experiments are necessary to verify their involvement in the Tween-degrading lipolytic activity.

### 3.6. Taxonomic Description of New Species

Description of *Fanglaniella lipolytica* gen. nov. sp. nov.

*Fanglaniella* A.H. Li & Y.J. Liao, gen. nov.

Fungal Names: FN 573040

Etymology: Fang,.lan.i.el’la. N.L. fem. n. *Fanglaniella*, named in honor of Mr. Fanglan Dai for his outstanding contributions to mycology in China.

Type species: *Fanglaniella lipolytica*

*Fanglaniella lipolytica* A.H. Li & Y.J. Liao, sp. nov.

Fungal Names: FN 573041

Etymology: li.po.ly’ti.ca. Gr. neut. n. *lipos*, fat; Gr. masc. adj. *lytikos*, dissolving; N.L. fem. adj. *lipolytica*, dissolving fat or lipid.

Culture characteristics: After growth on PDA for 3 days at 25 °C, cells were ovoid to ellipsoidal, 2.7–4.1 µm × 3.6–5.4 µm, and occurred singly or in pairs. Budding was polar (Figure 2). After 1 month at 25 °C in YM broth cultures, a sediment and a ring were present. On YM agar, after 7 days at 25 °C, the streak culture was yellowish-cream, butyrous, glistening, and smooth with the entire margin. On Dalmau plates after 2 weeks at 25 °C, on CMA, pseudohyphae were not observed. Sexual structures were not observed on 5% MEA, CMA, PDA, YCB, and YM agar. On CMA, ballistoconidia were not produced.

The physiological characteristics of the new species and the other five novel species were listed in Table 3.

The holotype, CGMCC 2.6218^T^, was isolated from the flowers of *Prunus persica ‘Albo-plena’* collected from the Beijing Olympic Forest Park, China, and has been deposited in a metabolically inactive state in the CGMCC, Beijing, China. The ex-type culture has been deposited in the Japan Collection of Microorganisms (JCM), Koyadai, Japan, as JCM 38231. The GenBank/EMBL/DDBJ accession numbers for the 26S rRNA gene D1/D2 domain and the ITS sequence of strain CGMCC 2.6218^T^ are PV981755 and PX225953. The raw genome data of CGMCC 2.6218^T^ have been deposited in GenBank under the BioSample accession numbers SAMN52392630 and the BioProject accession number PRJNA1338043. This Whole Genome Shotgun project has been deposited at DDBJ/ENA/GenBank under the accession JBTBFO000000000.

Description of *Polychromogenomyces tardus* gen. nov. sp. nov.

*Polychromogenomyces* A.H. Li & Y.J. Liao, sp. nov.

Fungal Names: FN 573042

Etymology: Po.ly.chro.mo.ge.no.my’ces. Gr. masc. adj. *polys*, many; Gr. neut. n. *chroma*, color; Gr. ind. v. *gennao*, produce; Gr. masc. n. *mykes*, fungus; N.L. masc. n. *Polychromogenomyces*, a yeast that produces many pigments.

Type species: *Polychromogenomyces tardus*

*Polychromogenomyces tardus* A.H. Li & Y.J. Liao, sp. nov.

Fungal Names: FN 573043

Etymology: tar’dus. L. masc. adj. *tardus*, slow, since it grows more slowly than the other five species.

Culture characteristics: After growth on PDA for 3 days at 25 °C, cells were subglobose, ellipsoidal, and oval, 3.4–5.1 µm × 4.8–6.5 µm, single or in pairs. Budding was polar (Figure 2). After 1 month at 25 °C in YM broth cultures, a sediment was present. On YM agar, after 7 days at 25 °C, the streak culture was pink, butyrous, smooth, and glistening. The margin was entire. Pseudohyphae were not produced on CMA slide cultures after 2 weeks at 25 °C. Sexual structures were not observed on 5% MEA, CMA, PDA, YCB, and YM agar. On CMA, ballistoconidia were not produced (Table 3).

The holotype, CGMCC 2.8784^T^, was isolated from the flowers of *Prunus persica ‘Albo-plena’* collected from the Beijing Olympic Forest Park, China, and has been deposited in a metabolically inactive state in the CGMCC, Beijing, China. The ex-type culture has been deposited in the Japan Collection of Microorganisms (JCM), Koyadai, Japan, as JCM 38239. The GenBank/EMBL/DDBJ accession numbers for the 26S rRNA gene D1/D2 domain and the ITS sequence of strain CGMCC 2.8784^T^ are PV981763 and PX225958. The raw genome data of CGMCC 2.8784^T^ have been deposited in GenBank under the BioSample accession numbers SAMN52395896 and the BioProject accession number PRJNA1338122. This Whole Genome Shotgun project has been deposited at DDBJ/ENA/GenBank under the accession JBTBFQ000000000.

*Pseudotremella jasmini* A.H. Li & Y.J. Liao, sp. nov.

Fungal Names: FN 573036

Etymology: jas. mi’ni. L. gen. n. *jasmini*, of *Jasminum*, since it has been isolated from the flower of *Jasminum nudiflorum*.

Culture characteristics: After growth on PDA for 3 days at 25 °C, the cells were ellipsoidal to oval, 2.2–3.7 µm × 2.9–4.6 µm, and occurred singly or as budded pairs. Budding was polar (Figure 2). After 1 month at 25 °C in YM broth cultures, a sediment and a ring were present. On YM agar, after 7 days at 25 °C, the streak culture was whitish-cream, butyrous, glistening, and smooth with the entire margin. Pseudohyphae were not produced on CMA slide cultures after 2 weeks at 25 °C. Sexual structures were not observed on 5% MEA, CMA, PDA, YCB, and YM agar. On CMA, ballistoconidia were not produced (Table 3).

Physiologically, *Pseudotremella jasmini* differed from its closest relatives *Pse. lacticolor* in its ability to assimilate nitrate, ethylamine hydrochloride, and cadaverine, and its inability to assimilate soluble starch, erythritol, and citrate. Additionally, *Pseudotremella jasmini* could grow in a medium supplemented with 50% glucose and at 35 °C, conditions under which *Pse. lacticolor* failed to grow.

The holotype, CGMCC 2.6068^T^, was isolated from the flowers of *Jasminum nudiflorum* collected from the Beijing Olympic Forest Park, China, and has been deposited in a metabolically inactive state in the CGMCC, Beijing, China. The ex-type culture has been deposited in the Japan Collection of Microorganisms (JCM), Koyadai, Japan, as JCM 38229. The GenBank/EMBL/DDBJ accession numbers for the 26S rRNA gene D1/D2 domain and the ITS sequence of strain CGMCC 2.6068^T^ are PV981753 and PX225951. The raw genome data of CGMCC 2.6068^T^ have been deposited in GenBank under the BioSample accession numbers SAMN52392628 and the BioProject accession number PRJNA1338041. This Whole Genome Shotgun project has been deposited at DDBJ/ENA/GenBank under the accession JBTBFM000000000.

*Teunia pruni* A.H. Li & Y.J. Liao, sp. nov.

Fungal Names: FN 573037

Etymology: pru’ni. L. gen. n. *pruni*, of *Prunus*, since it has been isolated from the flower of *Prunus persica ‘Kikumomo’*.

Culture characteristics: After growth on PDA for 3 days at 25 °C, cells were ellipsoidal and oval, 3.1–4.8 µm × 4.1–6.2 µm, single or in pairs. Budding was polar (Figure 2). After 1 month at 25 °C in YM broth cultures, a sediment was present. On YM agar, after 7 days at 25 °C, the streak culture was whitish-cream, butyrous, smooth, and glistening. The margin was entire. In Dalmau plate culture on CMA, pseudohyphae were not formed. Sexual structures were not observed on5% MEA, CMA, PDA, YCB, and YM agar. On CMA, ballistoconidia were not produced (Table 3).

Physiologically, *Teunia pruni* differed from its close relatives *Teunia heritierae*, *Teunia betulicola*, and *Teunia parabetulicola* by being able to assimilate galactose, melibiose, raffinose, ribitol, DL-lactate, and citrate, but unable to assimilate inulin. In addition, *Teunia pruni* could grow at 32 °C, whereas the aforementioned relatives could not.

The holotype, CGMCC 2.8783^T^, was isolated from the flowers of *Prunus persica ‘Kikumomo’* collected from the Beijing Olympic Forest Park, China, and has been deposited in a metabolically inactive state in the CGMCC, Beijing, China. The ex-type culture has been deposited in the Japan Collection of Microorganisms (JCM), Koyadai, Japan, as JCM 38238. The GenBank/EMBL/DDBJ accession numbers for the 26S rRNA gene D1/D2 domain and the ITS sequence of strain CGMCC 2.8783^T^ are PV981762 and PX225957. The raw genome data of CGMCC 2.8783^T^ have been deposited in GenBank under the BioSample accession numbers SAMN51794166 and the BioProject accession number PRJNA1333365. This Whole Genome Shotgun project has been deposited at DDBJ/ENA/GenBank under the accession JBTBFJ000000000.

*Kurtzmanomyces yulaniae* A.H. Li & Y.J. Liao, sp. nov.

Fungal Names: FN 573038

Etymology: yu.la’ni.ae. N.L. gen. n. *yulaniae*, of *Yulania*, since it has been isolated from the flower of *Yulania liliiflora*.

Culture characteristics: After growth on PDA for 3 days at 25 °C, cells were subglobose, ellipsoidal, and globose, 2.6–5.0 µm × 3.2–5.4 µm, single or in pairs. Budding was polar (Figure 2). After 1 month at 25 °C in YM broth cultures, a sediment and a ring were present. On YM agar, after 7 days at 25 °C, the streak culture was whitish-cream to pale yellowish-cream, butyrous, smooth, and with an entire margin. Pseudohyphae were not produced on CMA slide cultures after 2 weeks at 25 °C. Sexual structures were not observed on 5% MEA, CMA, PDA, YCB, and YM agar. On CMA, ballistoconidia were not produced (Table 3).

Physiologically, *Kurtzmanomyces yulaniae* differed from its closest relative *K. nectairei* in its ability to assimilate galactose, sucrose, maltose, cellobiose, lactose, raffinose, melezitose, soluble starch, D-ribose, L-arabinose, glycerol, erythritol, methyl-α-D-glucoside, and salicin, and its inability to assimilate DL-lactate. Furthermore, *Kurtzmanomyces yulaniae* could grow in a vitamin-free medium, while *K. nectairei* could not.

The holotype, CGMCC 2.8812^T^, was isolated from the flowers of *Yulania liliiflora* collected from the Beijing Olympic Forest Park, China, and has been deposited in a metabolically inactive state in the CGMCC, Beijing, China. The ex-type culture has been deposited in the Japan Collection of Microorganisms (JCM), Koyadai, Japan, as JCM 38241. The GenBank/EMBL/DDBJ accession numbers for the 26S rRNA gene D1/D2 domain and the ITS sequence of strain CGMCC 2.8812^T^ are PV981765 and PX225959. The raw genome data of CGMCC 2.8812^T^ have been deposited in GenBank under the BioSample accession numbers SAMN52392631 and the BioProject accession number PRJNA1338044. This Whole Genome Shotgun project has been deposited at DDBJ/ENA/GenBank under the accession JBTBFP000000000.

*Trigonosporomyces otomorphus* A.H. Li & Y.J. Liao, sp. nov.

Fungal Names: FN 573039

Etymology: o.to.mor’phus. Gr. neut. n. *ous* (gen. *otos*), the ear; Gr. fem. n. *morphè*, shape; N.L. masc. adj. *otomorphus*, ear-shaped, from its colony morphology.

Culture characteristics: After growth on PDA for 3 days at 25 °C, the cells were ellipsoidal or cylindrical, 1.3–2.1 µm × 5.2–19.1 µm, occasionally irregular, and occurred singly, in pairs, or short chains (Figure 2). After 1 month at 25 °C in YM broth cultures, a sediment and a ring were present. On YM agar, after 7 days at 25 °C, the streak culture was whitish-cream, crispulate, raised, restricted, and dull. The margin was lobate. After culturing the strain for 2 weeks in a Dalmau plate on CMA, pseudohyphae were observed. Sexual structures were not observed on YCB, 5% MEA, YM, PDA, and CMA. Ballistoconidia were not produced on CMA (Table 3).

Physiologically, *Trigonosporomyces otomorphus* differed from its closest relative *Trigonosporomyces hylophilus* in its ability to assimilate sucrose, citrate, inositol, and nitrate, and its inability to assimilate L-sorbose and D-ribose. In addition, *Trigonosporomyces otomorphus* could grow in a vitamin-free medium and at 37 °C, while *Trigonosporomyces hylophilus* could not.

The holotype, CGMCC 2.6214^T^, was isolated from the flowers of *Prunus persica ‘Albo-plena’* collected from the Beijing Olympic Forest Park, China, and has been deposited in a metabolically inactive state in the CGMCC, Beijing, China. The ex-type culture has been deposited in the Japan Collection of Microorganisms (JCM), Koyadai, Japan, as JCM 38230. The GenBank/EMBL/DDBJ accession numbers for the 26S rRNA gene D1/D2 domain and the ITS sequence of strain CGMCC 2.6214^T^ are PV981754 and PX225952. The raw genome data of CGMCC 2.6214^T^ have been deposited in GenBank under the BioSample accession numbers SAMN52392629 and the BioProject accession number PRJNA1338042. This Whole Genome Shotgun project has been deposited at DDBJ/ENA/GenBank under the accession JBTBFN000000000.

## 4. Discussion

Flowers serve as a common habitat for yeasts, with the majority of isolates originating from floral nectar, a niche characterized by high cellular density [43,44] yet low species richness [44,45]. Over the past decade, investigations into yeast diversity associated with flowers and their insect visitors have led to the description of more than 50 novel species. These taxa have predominantly belonged to the genera *Wickerhamiella* [46,47,48,49], *Metschnikowia* [50,51,52,53], *Starmerella* [54,55,56,57,58,59], *Kodamaea* [60,61], *Naganishia* [62], *Fonsecazyma* [63], and *Teunia* [64]. In the present study, host-specialized endemic species dominated floral yeast communities across all sampled plants, while shared generalist yeasts only occurred as minor accessory taxa, demonstrating the strong host selectivity of flower-associated yeasts, consistent with previous reports [17]. Furthermore, our results revealed anthropogenic influences on floral yeast assemblages within the Beijing Olympic Forest Park, supported by the recovery of *Aureobasidium* and *Cryptococcus*. Meanwhile, typical nectar-specialized yeast genera, such as *Metschnikowia* and *Starmerella*, were consistently detected at low relative abundances.

Nevertheless, the ecological and evolutionary mechanisms underlying this unique community structure with high density and low richness remain poorly understood. We suppose that nectar, as a high-sugar and hyperosmotic extreme habitat, serves as a powerful environmental filter, allowing only a limited set of yeast lineages with specific physiological adaptations, such as tolerance to elevated osmotic pressure and high sugar concentrations, to successfully colonize. To support this hypothesis, four of the six strains examined in this study demonstrated growth under 60% glucose conditions, including *Fanglaniella lipolytica* CGMCC 2.6218ᵀ, *jasmini* CGMCC 2.6068ᵀ, *Teunia pruni* CGMCC 2.8783ᵀ, and *Trigonosporomyces otomorphus* CGMCC 2.6214ᵀ.

Furthermore, to successfully colonize floral niches, to compete with other microbes, and to sustain their mutualistic associations with pollinator insects within the nutritionally unbalanced nectar microenvironment, these specialized yeasts likely deploy diverse nutrient acquisition strategies. Central to such adaptations is the secretion of extracellular enzymes to break down and utilize complex organic compounds in the environment. This functional capacity not only broadened their nutritional niche but reinforced their key ecological role in floral systems. We hypothesized that extracellular enzymes, including lipolytic enzymes and proteases, played distinct roles within the nectar microecosystem. For instance, lipolytic enzymes not only catalyzed the hydrolysis of lipids, but drove reactions crucial for synthesizing volatile aromatic esters, key compounds that attract pollinators. In this way, lipolytic enzymes might be involved in mediating the tripartite interactions among yeast, plants, and insects.

In the present study, all six novel species were confirmed to exhibit lipolytic activities. Besides validation via physiological experiments, genomic analyses further confirmed these metabolic capabilities. Moreover, genes encoding triacylglycerol lipases (TGL) and hormone-sensitive lipases (HSL) were also annotated in these novel strains. Collectively, these findings indicated that the long-chain fatty acid ester hydrolysis capacity of these strains was potentially attributed to the combined effects of the CE 10 family, TGL and HSL.

Currently, the efficient lipolytic producers reported in the literature are predominantly concentrated in genera such as *Candida*, *Cryptococcus*, *Rhodotorula*, *Yarrowia*, *Pichia*, and *Saccharomyces* [65,66]. Yeasts capable of producing lipolytica activity are predominantly reported in genera such as *Hanseniaspora*, *Candida*, *Metschnikowia*, *Pichia*, *Wickerhamomyces*, and *Torulaspora* [67]. Our findings indicate that the ability to produce lipolytic enzyme may be more widespread among yeasts than previously recognized. Furthermore, extracellular protease activity was detected in *F. lipolytica*, *Tri. otomorphus*, and *Pse. Jasmini*, though the activity was relatively weak. It is reported that protease-producing yeasts were mainly found in genera such as *Wickerhamomyces*, *Candida*, *Metschnikowia*, *Yarrowia*, *Rhodotorula*, and *Cryptococcus* [68,69]. The enzyme activity profiles of these novel species revealed a highly specialized catabolic phenotype, in which lipid and ester substrates were preferentially acquired and utilized rather than proteins. This metabolic strategy allowed the yeasts to efficiently exploit unique nutritional resources within the nectar microhabitat while mitigating adverse effects on floral hosts, consequently stabilizing the mutualistic tripartite interaction among flowers, yeasts, and pollinators. Further research dissecting the functional roles of yeast extracellular enzymes within floral microecosystems is of great value.

## 5. Conclusions

This study focused on a variety of flower samples which harbor diverse microbial taxa, including ecologically significant yeast species. A substantial number of flower samples were collected from the Beijing Olympic Forest Park. Yeast diversity within these collected samples was analyzed using the dilution-to-spread plating method, leading to the identification of 69 yeast species, which included two novel genera and four novel species. Furthermore, traditional yeast identification was performed for ten newly isolated strains representing six novel species. The results revealed the diversity of floral yeasts in the Beijing Olympic Forest Park. An extracellular enzyme activity assessment revealed that lipolytic activities were detected in all six novel species. Further genomic analysis demonstrated that these lipolytic activities potentially correlated with the synergistic functions of the CE10 family of CAZymes, alongside genes encoding triacylglycerol lipase (TGL) and hormone-sensitive lipase (HSL).

## Figures and Tables

**Table 1:** Genome sequence data of the type strains of six novel species.

Strains	Accession Number	Genome Size (Mb)	Sequence Depth	GC Content	Gene No.
CGMCC 2.6068	JBTBFM000000000	21.1	108×	52%	7371
CGMCC 2.6214	JBTBFN000000000	12.1	454×	52.5%	4553
CGMCC 2.6218	JBTBFO000000000	17.6	327×	55.5%	4931
CGMCC 2.8783	JBTBFJ000000000	20.8	282×	55.5%	6415
CGMCC 2.8784	JBTBFQ000000000	23.5	218×	54.5%	6495
CGMCC 2.8812	JBTBFP000000000	18.2	360×	56.5%	7842

**Table 2:** Genes potentially linked to lipolytic activities in genomes of these six novel species.

Gene ID	Gene Name	Swiss-Prot Description
***Fanglaniella lipolytica*** **CGMCC 2.6218**		
gene2153	lip	Uncharacterized protein C57A7.05
gene2612	lip	Lipase B
gene3107	TCL	Triacylglycerol lipase 2
gene2424	HSL	Hormone-sensitive lipase
***Polychromogenomyces tardus*** **CGMCC 2.8784**		
gene3033	TCL	Triacylglycerol lipase 2
gene5698	TCL	Triacylglycerol lipase 2
gene2736	HSL	Hormone-sensitive lipase
***Pseudotremella jasmini*** **CGMCC 2.6068**		
gene2700	TCL	Triacylglycerol lipase 2
gene5178	TCL	Triacylglycerol lipase 2
gene5866	HSL	Hormone-sensitive lipase
***Teunia pruni*** **CGMCC 2.8783**		
gene2448	TCL	Triacylglycerol lipase 2
gene2967	TCL	Triacylglycerol lipase 2
***Kurtzmanomyces yulaniae*** **CGMCC 2.8812**		
gene5741	TCL	Triacylglycerol lipase 2
gene6514	TCL	Triacylglycerol lipase 2
gene4040	LIPE	AB hydrolase superfamily protein C1039.03
***Trigonosporomyces otomorphus*** **CGMCC 2.6214**		
gene0310	TCL	Triacylglycerol lipase 2
gene2453	TCL	Triacylglycerol lipase 2
gene3248	HSL	Hormone-sensitive lipase

**Table 3:** Physiological and biochemical characteristics of the six novel taxa.

	*F. lipolytica*	*Pol. tardus*	*Pse.* *jasmini*	*Teu. pruni*	*K. yulaniae*	*Tri. otomorphus*
**Fermentation of**						
Glucose	−	−	−	−	−	−
Galactose	−	−	−	−	−	−
Sucrose	−	−	−	−	−	−
Maltose	−	−	−	−	−	−
Lactose	−	−	−	−	−	−
Raffinose	−	−	−	−	−	−
**Assimilation of carbon compounds**						
Glucose	+	+	+	+	+	+
Galactose	−	+	+	w	w	−
L-sorbose	−	+	+	+	−	−
Sucrose	+	w	+	+	+	w
Maltose	+	w	+	+	+	−
Cellobiose	w	+	+	+	+	−
Trehalose	+	+	+	+	+	−
Lactose	−	−	w	+	+	−
Melibiose	−	w	+	+	−	−
Raffinose	+	w	+	+	+	−
Melezitose	+	w	+	+	+	−
Inulin	−	w	w	−	−	−
Soluble starch	+	w	−	+	+	−
D-Xylose	−	+	+	+	+	−
D-Ribose	−	+	w	+	w	−
L-Arabinose	−	−	+	+	+	−
L-Rhamnose	−	w	+	+	−	−
Methanol	−	w	−	−	−	−
Ethanol	−	−	+	+	+	+
Glycerol	−	+	+	+	w	+
Erythritol	−	−	−	−	w	−
Ribitol	−	−	+	+	+	−
Galactitol	−	−	+	+	−	−
D-Mannitol	w	+	+	+	+	+
Sorbitol	w	+	+	+	w	+
Methyl-α-D-Glucoside	+	w	+	+	+	−
Salicin	+	+	+	+	w	−
DL-Lactate	w	−	−	+	−	−
Succinic acid	+	−	w	+	w	+
Citrate	+	−	−	w	−	w
Inositol	−	−	+	−	−	+
**Assimilation of nitrogen compounds**						
Nitrate	+	w	w	+	+	+
Nitrite	−	−	−	−	−	−
L-Lysine	+	+	+	+	+	+
Ethylamine hydrochloride	+	w	w	+	+	−
Cadaverine	+	+	+	+	+	w
Creatine	+	w	w	+	w	w
Creatinine	+	+	+	+	+	+
**Other tests**						
Vitamin-free	+	+	+	+	+	+
50% glucose	+	−	+	+	−	+
60% glucose	+	−	+	+	−	w
10% NaCl + 5% glucose	+	−	+	w	−	−
Urease activity	+	+	+	+	+	+
Diazonium Blue B	+	+	+	+	+	+
**Temperature tolerance**						
30 °C	+	w	+	+	w	+
32 °C	+	−	+	+	−	+
35 °C	+	−	w	−	−	+
37 °C	−	−	−	−	−	w
40 °C	−	−	−	−	−	−

Note: +, positive; −, negative; w, weakly positive.

## Data Availability

All gene and genome sequences generated in this study are available in GenBank under the accession numbers provided, with all additional data included in the manuscript and its supplements. The accession numbers for the LSU rRNA gene D1/D2 domains and ITS region of strain CGMCC 2.6218, CGMCC 2.8784, CGMCC 2.6068, CGMCC 2.6214, CGMCC 2.8783, and CGMCC 2.8812 are PV981755 and PX225953, PV981763 and PX225958, PV981753 and PX225951, PV981754 and PX225952, PV981762 and PX225957, and PV981765 and PX225959, respectively. Their genome accession numbers are JBTBFO000000000, JBTBFQ000000000, JBTBFM000000000, JBTBFN000000000, JBTBFJ000000000, and JBTBFP000000000, respectively.

## Supplementary Materials

The following supporting information can be downloaded at: https://www.mdpi.com/article/10.3390/jof12070521/s1, Figure S1: Distribution Map of Sampling Sites; Figure S2: Neighbor-joining phylogenetic tree showing the phylogenetic position of six novel species based on the D1/D2 domain and ITS region sequence; Figure S3: Protease activity after two weeks of growth: (a) *Fanglaniella lipolytica* CGMCC 2.6218; (b) *Trigonosporomyces otomorphus* CGMCC 2.6214; (c) *Pseudotremella jasmini* CGMCC 2.6068. Table S1: The D1D2 domain and ITS region identification results of the novel strains; Table S2: Taxa used for the phylogenetic analysis, strain information, and GenBank accession numbers. The newly generated sequences in the context of the present study are indicated in bold; Table S3: Virulence factors presented in the genome of the novel yeast strains; Table S4: Antibiotic resistance genes presented in the genome of these novel yeast strains. Data type: fasta. All alignments used for phylogenetic analysis in this research.

## Abbreviations

The following abbreviations are used in this manuscript:

CGMCC	China General Microbiological Culture Collection Center
CMA	Corn meal agar
HSL	Hormone-sensitive lipase
ITS	Internal transcribed spacer
LSU	Large subunit
MEA	Malt extract agar
ML	Maximum likelihood
PDA	Potato dextrose agar
SEM	Scanning electron microscopy
TGL	Triacylglycerol lipase
YM	Yeast extract–malt extract
