# Supplementary material for: Taxonomic Identification of Two Novel Genera and Four Novel Species of Lipolytic Floral-Associated Yeasts
Source: J Fungi (Basel). 2026 Jul 15;12(7):521. doi: 10.3390/jof12070521 (PMC13413130; doi:10.3390/jof12070521)
Supplement: Supplementary file 1 [file jof-12-00521-s001.zip › Table S1.pdf]

Table S1. The D1D2 domain and ITS region identification results of the novel strains

Strains	Accession No. of D1D2	Closest species	Similarity	Accession No.	Accession No. of ITS	Closest species	Similarity	Accession No.
CGMCC 2.6218	PV981755	*Yurkovia longicylindrica*	94.46%	MK050441	PX225953	*Pseudohyphozyma hydrangeae*	86.25%	MK050443
CGMCC 2.8784	PV981763	*Symmetrospora foliicola*	98.77%	NG058410	PX225958	*Sakaguchia lamellibrachiae*	89.95%	AB025999
CGMCC 2.6068	PV981753	*Pseudotremella lacticolor*	96.47%	NG060058	PX225951	*Pseudotremella lacticolor*	88.33%	NR158875
CGMCC 2.6214	PV981754	*Trigonosporomyces hylophilus*	94.66%	NG058423	PX225952	*Trigonosporomyces hylophilus*	89.05%	KY105766
CGMCC 2.8783	PV981762	*Teunia heritierae*	98.75%	OP470194	PX225957	*Teunia heritierae*	97.35%	NR200473
CGMCC 2.8812	PV981765	*Kurtzmanomyces nectairei*	96.63%	KY108195	PX225959	*Kurtzmanomyces nectairei*	82.88%	NR073266
