# Supplementary material for: Taxonomic Identification of Two Novel Genera and Four Novel Species of Lipolytic Floral-Associated Yeasts
Source: J Fungi (Basel). 2026 Jul 15;12(7):521. doi: 10.3390/jof12070521 (PMC13413130; doi:10.3390/jof12070521)
Supplement: Supplementary file 1 [file jof-12-00521-s001.zip › Table S2 .pdf]

Table S2: Taxa used for the phylogenetic analysis, strains information and GenBank accession numbers. The newly generated sequences in the context of the present study are indicated in bold.

**Species**	**Strains**	**ITS**	**D1/D2**	**References**
**Figure 2a**				
Fanglaniella lipolytica gen. nov. sp. nov.	**CGMCC 2.6218**	**PX225953**	**PV981755**	**This study**
Trigonosporomyces otomorphus sp. nov.	**CGMCC 2.6214**	**PX225952**	**PV981754**	**This study**
Aurantiosporium scleriae	SOMF 30248	MT636671	MT636661	Vánky (2002)
Bauerago abstrusa	HUV 18526	DQ238719	EF621955	(genbank 2025)
Bannozyma arctica	JCM 13290	NR153630	NG058612	Vishniac and Takashima (2010)
Bannozyma yamatoana	CBS 7243	NR167940	NG070544	(genbank 2025)
Begerowomyces foliicola	CGMCC 2.3164	NR174036	MK050394	Li et al. (2020)
Chrysozyma cylindrica	CGMCC 2.3455	NR174797	MK050439	Li et al. (2020)
Chrysozyma griseoflava	CBS 7284	NR073303	NG058746	Sun et al. (2025)
Chrysozyma sambuci	CGMCC 2.2618	NR174793	MK050431	Li et al. (2020)
Chrysozyma sorbariae	CGMCC 2.2768	NR174795	MK050435	Li et al. (2020)
Chrysozymaceae sp.	isolate NB124-2	OQ434130	OQ434130	(genbank 2025)
Colacogloea aletridis	CGMCC 2.2766	NR174802	MK050450	Li et al. (2020)
Colacogloea eucalyptica	CBS 8499	NR111685	KY106941	Pohl et al. (2011)
Colacogloea retinophila	CBS 8446	NR154830	NG058994	Thanh et al. (2004)
Colacogloea subericola	CECT 11976	NR137680	NG060065	Belloch et al. (2007)
Curvibasidium cygneicollum	CBS 4551	NR111077	NG042376	Sampaio et al. (2004)
Curvibasidium nothofagi	CBS 8166	NR073292	NG069005	(genbank 2025)
Fellozyma antarctica	CPCC 300301	OM980453	OM980542	Feng et al. (2025)
Fellozyma cerberi	DSM 102961	NR173420	FN428972	Yurkov et al. (2016)
Fellozyma inositophila	CBS 7310	NR073305	NG066180	Kachalkin (2022)
Fellozyma pinalis	VKM Y-2963	NR186990	OM666053	Golubev (2010)
Fellozyma telluris	DSM 102968	NR173419	FN428971	Yurkov et al. (2016)
Filobasidium dingjieense	CGMCC 2.5649	NR174759	MK050342	Li et al. (2020)
Fulvisporium restifaciens	no. 306	MT636672	MT636663	(genbank 2025)
Geastrum benitoi	MA-Fungi 87324	NR132912	NG060665	Zamora et al. (2015)
Geastrum kuharii	MA-Fungi 83795	NR132897	NG060656	Zamora et al. (2015)
Geastrum meridionale	MA-Fungi 87325	NR132896	NG060655	Zamora et al. (2015)
Geastrum papinuttii	MA-Fungi 83764	NR137855	NG060654	Zamora et al. (2015)
Glaciozyma watsonii	CBS 10986	NR155146	NG058294	Turchetti et al. (2011)

Hamamotoa lignophila	CBS 7109	NR111078	NG070402	(genbank 2025)
Hamamotoa singularis	CBS 5109	KY103496	KY107777	(genbank 2025)
Jaminaea lantanae	CGMCC 2.3529	MN901709	MN901709	Li et al. (2022)
Leucosporidium egoroviorum	KBP Y-6804	NR186988	OM038460	Kachalkin et al. (2023)
Leucosporidium fellii	CBS 7287	NR073276	AY512856	Yurkov et al. (2012)
Leucosporidium muscorum	CBS 6921	NR073286	KY108280	(genbank 2025)
Microbotryomycetes sp.	isolate JL201	OQ388269	OQ411308	(genbank 2025)
Microbotryozyma collariae	ATCC MYA-4666	NR111681	NG042600	Suh et al. (2012)
Microbotryozyma swertiae	CGMCC 2.3533	NR174790	MK050424	Li et al. (2020)
Oberwinklerozyma dicranopteridis	CGMCC 2.3441	NR174791	MK050426	Li et al. (2020)
Oberwinklerozyma yarrowii	CBS 7417	NR073328	NG058357	(genbank 2025)
Phenoliferia glacialis	CBS 10436	NR154358	NG058369	Margesin et al. (2007)
Phenoliferia psychrophenolica	CBS 10438	NR154289	NG066183	Margesin et al. (2007)
Pseudohyphozyma hydrangeae	CGMCC 2.2796	NR174801	MK050443	Li et al. (2020)
Pseudohyphozyma lulangensis	CGMCC 2.2612	NR174800	MK050442	Li et al. (2020)
Pseudohyphozyma pustula	CBS 6527	NR073288	NG058378	(genbank 2025)
Pseudoleucosporidium fasciculatum	CBS 8786	NR155333	KY108282	Li et al. (2020)
Rhodotorula alborubescens	CBS 482	NR153197	NG068967	Wei et al. (2025)
Rhodotorula dairenensis	CBS 4406	KY104735	NG057644	Nagahama et al. (2006)
Rhodotorula mucilaginosa	CBS 316	NR073296	KY109056	Wei et al. (2025)
Rhodotorula paludigena	CBS 6566	NR073265	KY109142	Wei et al. (2025)
Rhodotorula sphaerocarpa	CBS 5939	NR073269	KY109155	Wei et al. (2025)
Rhodotorula sp.	isolate S1-5	MW534871	MW534894	(genbank 2025)
Rhodosporidiobolus fluvialis	CBS 6568	NR077089	KY108963	Turchetti et al. (2018)
Rhodosporidiobolus fuzhouensis	CGMCC 2.4435	NR174784	MK050404	Li et al. (2020)
Rhodosporidiobolus lusitaniae	CBS 7604	NR077091	KY108964	Turchetti et al. (2018)
Rhodosporidiobolus platycladi	CGMCC 2.3118	NR174782	MK050401	Li et al. (2020)
Rhodosporidiobolus poonsookiae	CBS 9095	NR077115	NG058385	Takashima and Nakase (2000)
Robertozyma ningxiaensis	CGMCC 2.4451	NR174035	MK050392	Li et al. (2020)
Rosettozyma cystopteridis	CGMCC 2.2615	NR174038	MK050398	Li et al. (2020)
Rosettozyma petaloides	CGMCC 2.3446	NR174037	MK050395	Li et al. (2020)
Sampaiozyma ingeniosa	CBS 4240	NR111080	AF189934	(genbank 2025)
Sampaiozyma vanillica	CBS 7404	NR073315	NG058399	(genbank 2025)
Slooffia tsugae	CBS 5038	NR155840	KY109660	Yurkov et al. (2016)
Solicoccozyma aeria	CBS 155	NR155841	NG067315	Li et al. (2019)
Solicoccozyma zizaniae	DSM 104031	NR168770	MH718302	Yurkov and Kurtzman (2019)
Spencerozyma acididurans	CGMCC 2.5825	NR185556	NG228800	Yang et al. (2019)
Spencerozyma crocea	CBS 2029	NR103609	NG057723	Yang et al. (2019)

Spencerozyma pingqiaoensis	NYNU 178247	MG255729	MG255703	Chai et al. (2023)
Spencerozyma siamensis	DMKU13-2	LC315095	LC195008	Kaewkrajay and Limtong (2018)
Sporobolomyces blumeae	JCM 10212	NR137641	AY070007	Zhao et al. (2003)
Sporobolomyces johnsonii	CBS 5470	NR077090	NG042343	Zhao et al. (2003)
Sporobolomyces reniformis	CGMCC 2.5627	NR174786	MK050408	Li et al. (2020)
Sympodiomycopsis europaea	CGMCC 2.3119	MN901717	MN901717	Li et al. (2022)
Trigonosporomyces hylophilus	CBS 6226	NR154536	NG058423	(genbank 2025)
Ustilentyloma fluitans	RB 900	AY212990	AF009882	(genbank 2025)
Ustilentyloma graminis	CBS 6403	NR073285	NG057729	(genbank 2025)
Vonarxula javanica	CBS 5236	NR111079	KY110052	(genbank 2025)
Yunzhangia auriculariae	CBS 6379	NR073275	NG 058445	Feng et al. (2025)
Yunzhangia sonckii	CBS 6713	NR073326	NG057670	Feng et al. (2025)
Yurkovia castaneae	CGMCC 2.6909	NR200498	NG245475	Jiang et al. (2024)
Yurkovia longicylindrica	CGMCC 2.5603	NR174799	MK050441	Li et al. (2020)
Yurkovia mendeliana	PYCC 6884	NR147642	KU187888	Mašínova et al. (2017)
Yurkovia nerthusi	DSMZ 26788	NR173424	FN428970	Kachalkin et al. (2019)
**Figure 2b**				
**Species**	**Strains**	**ITS**	**D1/D2**	**Refernces**
***Polychromogenomyces tardus* gen. nov. sp. nov.**	**CGMCC 2.8784**	**PX225958**	**PV981763**	**This study**
Bannoa bischofiae	JCM 10338	NR153592	NG058609	Hamamoto et al. (2002)
Bannoa ellipsoidea	NYUN 2110396	OM014197	OM014195	Chai et al. (2023)
Bannoa foliicola	NYNU 208237	MW36554 1	MW365544	Chai et al. (2023)
Bannoa guamensis	CBS 16127	MK287350	MK255006	Parra et al. (2019)
Bannoa hahajimensis	JCM 10336	AB035897	NG042311	Hamamoto et al. (2002)
Bannoa macarangae	BRIP 28272	NR175756	NG079569	Tan et al. (2021)
Bannoa ogasawarensis	CBS 9038	AB035717	NG058699	Hamamoto et al. (2002)
Bannoa pseudofoliicola	NYUN 2110469	OM014200	OM014198	Chai et al. (2023)
Bannoa syzygii	CBS 9040	NR154870	NG058700	Hamamoto et al. (2002)
Bannoa tropicalis	CBS 16087	MK287360	MK255016	Parra et al. (2019)
Begerowomyces aurantius	CBS 16241	LC597190	LC597189	Guo et al. (2023)
Begerowomyces foliicola	CGMCC 2.3164	NR174036	MK050394	Li et al. (2020)
Buckleyzyma armeniaca	CBS 8076	NR073284	AF189920	Wang et al. (2015)
Buckleyzyma aurantiaca	CBS 317	NR073293	NG058618	Wang et al. (2015)
Buckleyzyma kluyveri-nielii	CBS 7168	NR073298	AF189988	Wang et al. (2015)
Buckleyzyma phyllomatis	CBS 7198	NR077097	NG066173	Wang et al. (2015)
Buckleyzyma pseudoaurantiaca	CGMCC 2.6834	OP470304	OP470208	Jiang et al. (2024)
Buckleyzyma salicina	CBS 6983	NR153595	NG058619	Wang et al. (2015)
Cystobasidium benthicum	CBS 9124	NR171726	NG059003	Nagahama et al. (2003)
Cystobasidium laryngis	CBS 2221	NR154833	KY107432	Fotedar et al. (2019)
Cystobasidium raffinophilum	CGMCC 2.3822	NR174780	MK050389	Li et al. (2020)

Cystobasidium ritchiei	CBS 12324	NR154854	LM644066	Yurkov et al. (2015)
Cystobasidium terricola	CGMCC 2.3823	NR174781	MK050390	Li et al. (2020)
Erythrobasidium elongatum	CBS 8080	NR073306	AF189983	Lu et al. (2024)
Erythrobasidium eucalypti	CBS 19176	PV133514	PV133516	Crous et al. (2025)
Erythrobasidium hasegawianum	JCM 1545	NR111008	AF131058	Lu et al. (2024)
Erythrobasidium leptospermi	BRIP 66853	NR175759	NG079571	Tan et al. (2021)
Erythrobasidium nanyangense	NYNU 208200	MW36236 0	MW362359	Lu et al. (2024)
Erythrobasidium penningtoniae	BRIP 76695a	PQ806954	PQ792645	Tan et al. (2024)
Erythrobasidium primogenitum	BRIP 72389e	NR182613	OP598058	Tan et al. (2022)
Erythrobasidium proteacearum	BRIP 66871	NR175760	NG079572	Tan et al. (2021)
Erythrobasidium turpiniae	NYUN 2110435	OM014199	OM014196	Lu et al. (2024)
Erythrobasidium yunnanense	CGMCC 2.2090	NR155098	NG059190	Wang et al. (2015)
Naohidea sebacea	CBS 8477	NR121324	NG042442	Lu et al. (2024)
Robertozyma ningxiaensis	CGMCC 2.4451	NR174035	MK050392	Li et al. (2020)
Sakaguchia cladiensis	CBS 10878	NR155829	NG066185	Fell et al. (2011)
Sakaguchia dacryoidea	CBS 6353	NR073323	NG058395	(genbank 2025)
Sakaguchia lamellibrachiae	CBS 9598	NR155767	NG058396	Nagahama et al. (2001)
Sakaguchia meli	CBS 10797	NR155775	AY158654	Libkind et al. (2010)
Sakaguchia melibiophila	JCM 8162	NR174883	NG079627	Li et al. (2020)
Sakaguchia oryzae	CBS 9745	KY105307	NG058397	Bai et al. (2004)
Symmetrospora clarorosea	CBS 14055	NR173721	NG075192	Haelewaters et al. (2020)
Symmetrospora coprosmae	CBS 7899	NR073317	AF189980	Haelewaters et al. (2020)
Symmetrospora eucalypti	BRIP 28188	NR174933	NG079568	Tan et al. (2021)
Symmetrospora foliicola	CBS 8075	NR073283	NG058410	Haelewaters et al. (2020)
Symmetrospora gracilis	CBS 71	NR073318	NG057671	Haelewaters et al. (2020)
Symmetrospora joanclarkeae	BRIP 76361a	PQ866158	PQ866160	Tan et al. (2024)
Symmetrospora marina	CBS 2365	NR073272	AF189944	Haelewaters et al. (2020)
Symmetrospora oryzicola	CBS 7228	NR073300	NG069416	Haelewaters et al. (2020)
Symmetrospora pini	CICC 33601	OQ851927	OQ851926	Wang et al. (2025)
Symmetrospora proteacearum	BRIP 45084	NR174932	MZ930245	Tan et al. (2021)
Symmetrospora pseudomarina	CBS 14057	NR171799	KJ701217	Haelewaters et al. (2020)
Symmetrospora rhododendri	CGMCC 2.2613	NR174779	MK050388	Li et al. (2020)
Symmetrospora salmoneus	CGMCC 2.6801	OM417187	OM417187	Wei et al. (2022)
Symmetrospora suhii	CBS 14094	NR171798	NG075154	Haelewaters et al. (2020)
Symmetrospora symmetrica	CBS 9727	NR158993	NG057632	Haelewaters et al. (2020)
Symmetrospora vermiculata	CBS 9092	NR077114	NG057633	Haelewaters et al. (2020)
**Figure 2c**				
**Species**	**Strains**	**ITS**	**D1/D2**	**References**
Pseudotremella jasmini sp. nov.	**CGMCC 2.6066**	**PV981752**	**PX225950**	**This study**
Pseudotremella jasmini sp. nov.	**CGMCC 2.6068**	**PV981753**	**PX225951**	**This study**
Bullera alba	CBS 500	NR111083	NG042387	Liao et al. (2025)

Bullera unica	CBS 8290	NR073256	NG058615	Liao et al. (2025)
Cryptococcus amylolentus	CBS 6039	NR111372	NG042481	Liao et al. (2025)
Papiliotrema aurea	CBS 318	NR130650	NG148937	Takashima et al. (2003)
Papiliotrema bandonii	CBS 9107	NR121465	KY108730	Sampaio et al. (2002)
Papiliotrema catalpae	CGMCC 2.6897	OP470271	OP470175	Jiang et al. (2024)
Papiliotrema fudaokuniae	BRIP 76370a	PQ279216	PQ279209	Gao et al. (2025)
Papiliotrema horticola	KBP Y-6685	NR182874	MW579431	Gao et al. (2025)
Papiliotrema japonica	CBS 2013	NR155613	NG057690	Gao et al. (2025)
Papiliotrema laurentii	CBS 139	AF410468	NG056281	Gao et al. (2025)
Papiliotrema leoncinii	CBS 13918	KP203864	KJ608554	Gao et al. (2025)
Papiliotrema miconiae	CBS 8358	AF444387	AF444698	Gao et al. (2025)
Papiliotrema nemorosa	CBS 9606	KY104472	KY108741	Gao et al. (2025)
Papiliotrema odontotermitis	CBS 14181	KU883277	KU883278	Gao et al. (2025)
Papiliotrema perniciosa	VKM Y-2905	NR137653	NG060063	Gao et al. (2025)
Papiliotrema plantarum	DMKU CP801	NR164566	LC370335	Into et al. (2018)
Papiliotrema rajasthanensis	CBS 10406	NR155678	NG058366	Gao et al. (2025)
Papiliotrema siamensis	DMKU SP85	NR155608	NG060062	Gao et al. (2025)
Papiliotrema tapputiae	BRIP 75038a	NR187102	NG229127	Gao et al. (2025)
Papiliotrema terrestris	CBS 10810	NR073350	NG058367	Gao et al. (2025)
Pseudotremella allantoinivorans	CBS 9604	NR137662	NG057722	Middelhoven (2004)
Pseudotremella hippophaes	CGMCC 2.6838	OP470285	OP470189	Jiang et al. (2024)
Pseudotremella lacticolor	CBS 10915	NR158875	NG060058	Satoh et al. (2013)
Pseudotremella lichenophila	CPCC 300091	OM980383	OM980472	Feng et al. (2025)
Pseudotremella moriformis	CBS 7810	NR155685	NG058379	Liu et al. (2015)
Pseudotremella navarinensis	RGM 3659	OQ448493	PQ362941	Pérez et al. (2024)
Pseudotremella nivalis	Dai20670	OL655317	OL677107	Liao et al. (2025)
Pseudotremella rhododendri	CGMCC 2.6854	OP470282	OP470186	Jiang et al. (2024)
Pseudotremella sp.	Wu489	PP982337	PP995645	(genbank 2025)
Pseudotremella sp.	Wu648	PP982338	PP995646	(genbank 2025)
Rhynchogastrema aquatica	CBS 12527	JN790616	KC171328	Valente et al. (2012)
Rhynchogastrema complexa	CBS 11570	NR111476	NG042525	Valente et al. (2012)
Rhynchogastrema coronatum	DSM 28188	LN870267	LN870267	Liao et al. (2025)
Rhynchogastrema fermentans	CBS 12399	NR155732	NG058388	Valente et al. (2012)
Rhynchogastrema glucofermentans	CBS 10381	NR119978	NG042404	Valente et al. (2012)
Rhynchogastrema nanyangensis	CBS 12474	NR166792	JN564592	Hui et al. (2012)
Rhynchogastrema noutii	CBS 8364	AF444391	AF444700	Valente et al. (2012)
Rhynchogastrema tunnelae	CBS 8024	NR111074	NG042390	Valente et al. (2012)
Rhynchogastrema visegradensis	CBS 12505	NR111591	NG058389	Valente et al. (2012)
**Figure 2d**				
**Species**	**Strains**	**ITS**	**D1/D2**	**References**
***Teunia pruni* sp. nov.**	**CGMCC 2.8779**	**PX225954**	**PV981759**	**This study**
***Teunia pruni* sp. nov.**	**CGMCC 2.8780**	**PX225955**	**PV981760**	**This study**

***Teunia pruni* sp. nov.**	**CGMCC 2.8781**	**PX225956**	**PV981761**	**This study**
***Teunia pruni* sp. nov.**	**CGMCC 2.8783**	**PX225957**	**PV981762**	**This study**
Cryptococcus amylolentus	CBS 6039	NR111372	NG042481	Liao et al. (2025)
Kwoniella bestiolae	CBS 10118	NR111373	NG042482	Liu et al. (2015)
Kwoniella botswanensis	CBS 12716	NR119822	HF545769	Guerreiro et al. (2013)
Kwoniella dejecticola	CBS 10117	NR111374	NG042483	Thanh et al. (2006)
Kwoniella dendrophila	CBS 6074	NR073257	AF189870	Chen et al. (2012)
Kwoniella endophytica	CBS 15359	NR170734	MH237945	Crous et al. (2018)
Kwoniella europaea	PYCC 6162	NR197462	AY167602	Guerreiro et al. (2013)
Kwoniella fici	DBVPG 10122	NR174641	NG078670	Kachalkin et al. (2019)
Kwoniella heveanensis	CBS 569	NR073210	NG058327	Metin et al. (2010)
Kwoniella hippophaes	CGMCC 2.6893	OP470262	OP470166	Jiang et al. (2024)
Kwoniella lonicerae	CGMCC 2.6883	OP470260	OP470164	Jiang et al. (2024)
Kwoniella mangrovensis	CBS 8507	NR073332	NG042391	Statzell-Tallman et al. (2008)
Kwoniella newhampshirensis	CBS 13917	NR156292	NG068520	Sylvester et al. (2015)
Kwoniella ovata	CGMCC 2.3439	NR174734	MK050289	Li et al. (2020)
Kwoniella pini	VKM Y-2958	NR111269	NG042453	Golubev et al. (2008)
Kwoniella shandongensis	CBS 12478	NR156242	JN160602	Chen et al. (2012)
Kwoniella shivajii	CBS 11374	NR165977	NG042515	Ravella et al. (2009)
Kwoniella sp.	7	MZ733389	MZ733296	(genbank 2025)
Kwoniella sp.	21A	MZ734342	MZ734329	(genbank 2025)
Kwoniella sp.	23	MZ734351	MZ734350	(genbank 2025)
Teunia acericola	NYUN 2111141	OM017172	OM017170	Guo et al. (2024)
Teunia betulae	CBS 13896	NR158392	NG068521	Li et al. (2020)
Teunia betulicola	CGMCC 2.7195	OP470263	OP470167	Jiang et al. (2024)
Teunia chimonanthi	CGMCC 2.6916	OP470289	OP470193	Jiang et al. (2024)
Teunia cuniculi	CBS 10309	NR137887	KY106982	Li et al. (2020)
Teunia globosa	CGMCC 2.5648	NR174733	MK050288	Li et al. (2020)
Teunia helanensis	CGMCC 2.4450	NR174732	MK050287	Li et al. (2020)
Teunia heritierae	CGMCC 2.6856	OP470290	OP470194	Jiang et al. (2024)
Teunia korlaensis	CGMCC 2.3835	NR174731	MK050286	Li et al. (2020)
Teunia littoralis	CGMCC 2.6461	OR577141	OR577145	Sun et al. (2025)
Teunia mussaendrae	NYNU 23232	OQ851888	OQ851887	Guo et al. (2024)
Teunia myricariae	CGMCC 2.6846	OP470287	OP470191	Jiang et al. (2024)
Teunia nitrariae	CGMCC 2.6797	OM417183	OM417183	Wei et al. (2022)
Teunia parabetulicola	CGMCC 2.6852	OP470264	OP470168	Jiang et al. (2024)
Teunia qingyuanensis	NYNU 22475	OP269841	OP269842	Guo et al. (2024)
Teunia quercus	CGMCC 2.6859	OP470291	OP470195	Jiang et al. (2024)
Teunia rhododendri	CGMCC 2.6896	OP470292	OP470196	Jiang et al. (2024)
Teunia rosae	CGMCC 2.5833	MK942578	MK942561	Wang et al. (2020)
Teunia rudbeckiae	CGMCC 2.5840	MK942577	MK942559	Wang et al. (2020)
Teunia siamensis	NBRC 114011	LC440108	LC420623	Khunnamwong et al. (2020)
Teunia tronadorensis	DSM 26994	NR165990	MF959620	Li et al. (2020)

Teunia turchettiae	KBP Y-6607	MT470198	MT470198	Kachalkin et al. (2024)
**Figure 2e**				
**Species**	**Strains**	**ITS**	**D1/D2**	**References**
***Kurtzmanomyces yulaniae* sp. nov.**	**CGMCC 2.8812**	**PX225959**	**PV981765**	**This study**
Ballistosporomyces bomiensis	CGMCC 2.2661	KP020108	KP020108	Han et al. (2016)
Ballistosporomyces changbaiensis	CBS 10124	NR186912	KP020105	Han et al. (2016)
Ballistosporomyces sasicola	CBS 7285	NR077095	NG058696	Wang et al. (2015)
Ballistosporomyces taupoensis	CBS 7898	NR077093	NG058698	Wang et al. (2015)
Ballistosporomyces xanthus	CBS 7513	NR153596	KY106152	Wang et al. (2015)
Bensingtonia ciliata	CBS 7514	NR073308	NG042363	Wang et al. (2012)
Bjerkandera centroamericana	voucher BRNM 771949	NR198012	KT305933	Westphalen et al. (2015)
Cabalodontia delicata	SP 512584	NR174056	MT849297	Westphalen et al. (2021)
Crittendenia heterodermiae	LPB Etayo 32711	OM521991	NG154010	Diederich et al. (2022)
Crittendenia hypotrachynae	PO Etayo 31093	NR184921	NG153921	Diederich et al. (2022)
Crittendenia kakouettae	BR van den Boom 58956	NR184966	NG154011	Diederich et al. (2022)
Crittendenia lecidellae	UBC Bjork 17999	MT520705	NG153922	Diederich et al. (2022)
Crittendenia lichenicola	E Coppins 21517	OM521994	OM521974	Diederich et al. (2022)
Crittendenia parvispora	BR DI047	NR184967	NG154012	Diederich et al. (2022)
Crittendenia physconiae	G AM1133	NR184922	NG153923	Diederich et al. (2022)
Crystallicutis damiettensis	ABS UN63	NR172196	MW508515	El-Gharabawy et al. (2021)
Cystobasidiopsis lactophilus	CBS 7527	NR073299	NG058726	Wang et al. (2025)
Cystobasidiopsis lophatheri	CBS 11272	NR144767	NG058719	Nakase et al. (2005)
Cystobasidiopsis nirenbergiae	TUB 019163	NR158377	NG058767	Bauer et al. (2009)
Fomitopsis serialis	GB KHL 12010	NR154676	JX109844	(genbank 2025)
Kondoa arboricola	CGMCC 2.2621	NR174767	MK050357	Li et al. (2020)
Kondoa chamaenerii	CGMCC 2.2652	NR174765	MK050354	Li et al. (2020)
Kurtzmanomyces guiyangensis	NYNU 23983	OR961459	OR958742	Wang et al. (2025)
Kurtzmanomyces insolitus	CBS 8377	NR073322	NG042355	Wang et al. (2025)
Kurtzmanomyces lichenum	DBVPG 8077T	PP781924	PP831005	Carolis et al. (2025)
Kurtzmanomyces nectairei	CBS 6405	NR073266	KY108195	Wang et al. (2025)
Kurtzmanomyces shapotouensis	CBS 12707	NR155216	NG057975	Zhang et al. (2013)
Kurtzmanomyces tardus	CBS 7421	NR073311	NG042357	Wang et al. (2025)
Lividopora facilis	H 7200351	OR262154	OR262154	Miettinen et al. (2023)
Microbotryozyma swertiae	CGMCC 2.3533	NR174790	MK050424	Li et al. (2020)
Mixia osmundae	JCM 22182	NR119614	NG068726	(genbank 2025)
Pseudoleucosporidium fasciculatum	CBS 8786	NR155333	KY108282	Li et al. (2020)

Ruinenia bangxiensis	CGMCC 2.3454	NR174775	MK050373	Li et al. (2020)
Ruinenia clavata	CGMCC 2.2318	NR155708	AY364839	Wang and Bai (2004)
Ruinenia diospyri	JCM 12157	NR144768	NG064303	Nakase et al. (2005)
Ruinenia dracophylli	CBS 7900	NR073320	AF189982	(genbank 2025)
Ruinenia fanjingshanensis	CGMCC 2.4542	NR174774	MK050372	Li et al. (2020)
Ruinenia lunata	CGMCC 2.4426	NR182793	KP020113	Li et al. (2020)
Ruinenia pyrrosiae	CBS 11273	NR144766	NG060783	Nakase et al. (2005)
Ruinenia rubra	CBS 7512	NR155733	AF189992	Nakase et al. (1989)
Slooffia tsugae	CBS 5038	NR155840	KY109660	(genbank 2025)
