# Supplementary material for: Taxonomic Identification of Two Novel Genera and Four Novel Species of Lipolytic Floral-Associated Yeasts
Source: J Fungi (Basel). 2026 Jul 15;12(7):521. doi: 10.3390/jof12070521 (PMC13413130; doi:10.3390/jof12070521)
Supplement: Supplementary file 1 [file jof-12-00521-s001.zip › Table S3 .pdf]

Table S3: Virulence factors presented in the genome of the novel yeast strains

Strains	Gene No.
Fanglaniella lipolytica gen. nov. sp. nov. CGMCC 2.6218	639
Polychromogenomyces tardus gen. nov. sp. nov. CGMCC 2.8784	693
Pseudotremella jasmini sp. nov. CGMCC 2.6068	693
Trigonosporomyces otomorphus sp. nov. CGMCC 2.6214	551
Teunia pruni sp. nov. CGMCC 2.8783	660
Kurtzmanomyces yulaniae sp. nov. CGMCC 2.8812	743
