# Supplementary material for: Taxonomic Identification of Two Novel Genera and Four Novel Species of Lipolytic Floral-Associated Yeasts
Source: J Fungi (Basel). 2026 Jul 15;12(7):521. doi: 10.3390/jof12070521 (PMC13413130; doi:10.3390/jof12070521)
Supplement: Supplementary file 1 [file jof-12-00521-s001.zip › Table S4 .pdf]

Table S4. Antibiotic resistance genes presented in the genome of these novel yeast strains

Strains / Drug Class	CGMCC 2.6218	CGMCC 2.8784	CGMCC 2.6068	CGMCC 2.6214	CGMCC 2.8783	CGMCC 2.8812
Pleuromutilin antibiotic	2	3	3	4	4	6
Fusidane antibiotic	4	3	2	3	1	2
Sulfonamide antibiotic	1	1	1	1	1	1
Macrolide antibiotic	7	8	5	3	10	14
Aminocoumarin antibiotic	5	8	4	4	3	5
Tetracycline antibiotic	21	29	21	16	28	25
Thioamide antibiotic	0	2	0	0	1	3
Disinfecting agents and antiseptics	11	12	17	9	14	25
Diaminopyrimidine antibiotic	1	2	1	3	2	5
Streptogramin antibiotic	1	3	4	2	3	5
Glycylcycline	0	1	1	0	1	2
Fluoroquinolone antibiotic	16	19	27	5	19	25
Cephalosporin	11	4	5	3	6	8
Phenicol antibiotic	3	4	7	6	6	5
Rifamycin antibiotic	6	10	7	2	5	8
Elfamycin antibiotic	2	2	4	2	1	1
Salicylic acid antibiotic	1	1	1	1	1	1
Oxazolidinone antibiotic	3	2	4	3	2	0
Isoniazid-like antibiotic	1	4	1	1	1	6
Streptogramin A antibiotic	1	2	2	1	2	3

Peptide antibiotic	15	9	12	9	13	15
Lincosamide antibiotic	3	3	5	3	6	5
Penam	17	3	8	5	11	15
Nitroimidazole antibiotic	5	5	7	3	4	10
Mupirocin-like antibiotic	3	3	3	4	2	2
Glycopeptide antibiotic	9	12	12	9	10	12
Streptogramin B antibiotic	0	2	2	0	0	0
Aminoglycoside antibiotic	3	1	1	2	0	2
Sulfone antibiotic	0	1	0	0	0	0
Pyrazine antibiotic	0	1	0	2	0	2
Antibacterial free fatty acids	0	0	2	0	0	1
Cephamycin	2	0	0	0	0	1
Nucleoside antibiotic	0	0	0	0	0	1
Bicyclomycin-like antibiotic	0	0	0	0	0	1
