# Supplementary figures and images for: Taxonomic Identification of Two Novel Genera and Four Novel Species of Lipolytic Floral-Associated Yeasts
Source: J Fungi (Basel). 2026 Jul 15;12(7):521. doi: 10.3390/jof12070521 (PMC13413130; doi:10.3390/jof12070521)

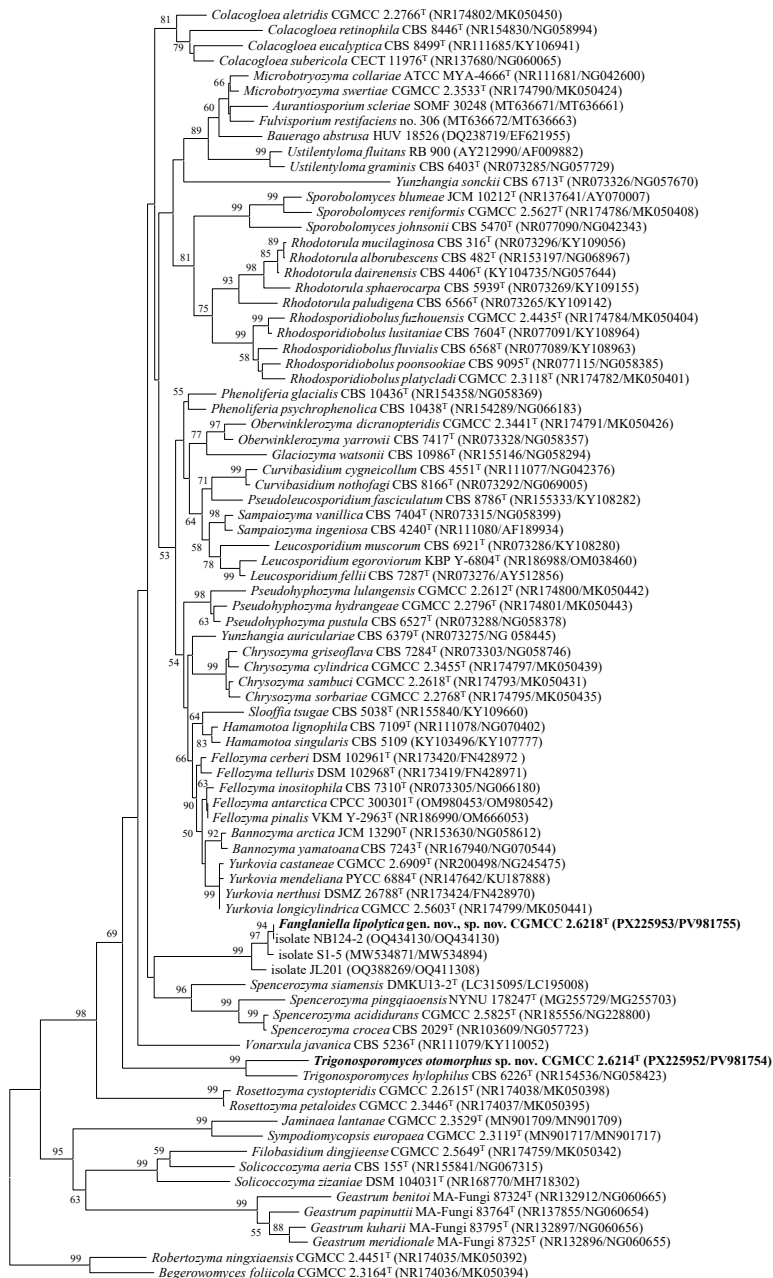

Supplement: Supplementary file 1 [file jof-12-00521-s001.zip › Figure S2/Figure S2a-- 2.6218,2.6214---NJ tree.pdf]

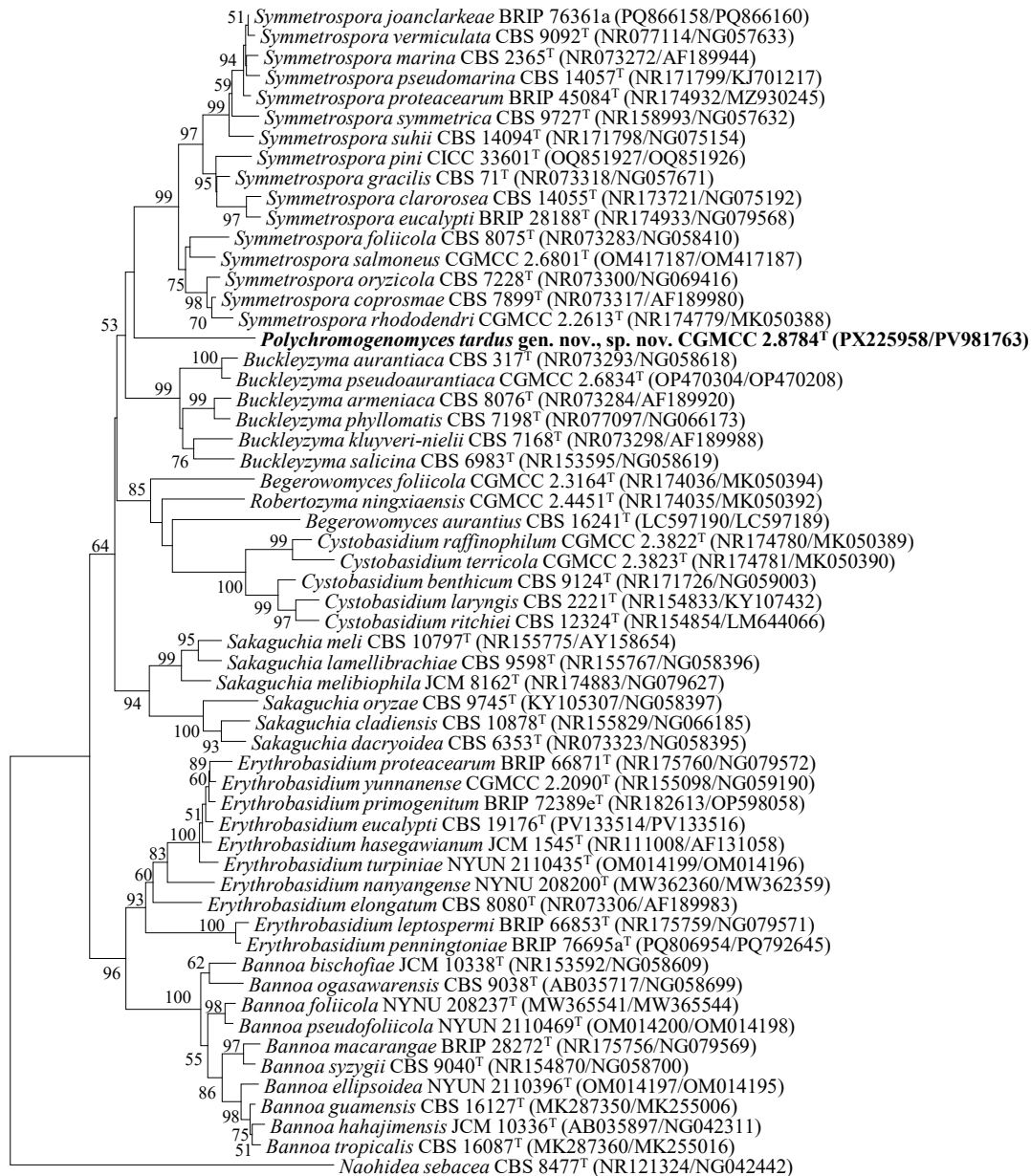

0.020

Supplement: Supplementary file 1 [file jof-12-00521-s001.zip › Figure S2/Figure S2b-- 2.8784---NJ tree.pdf]

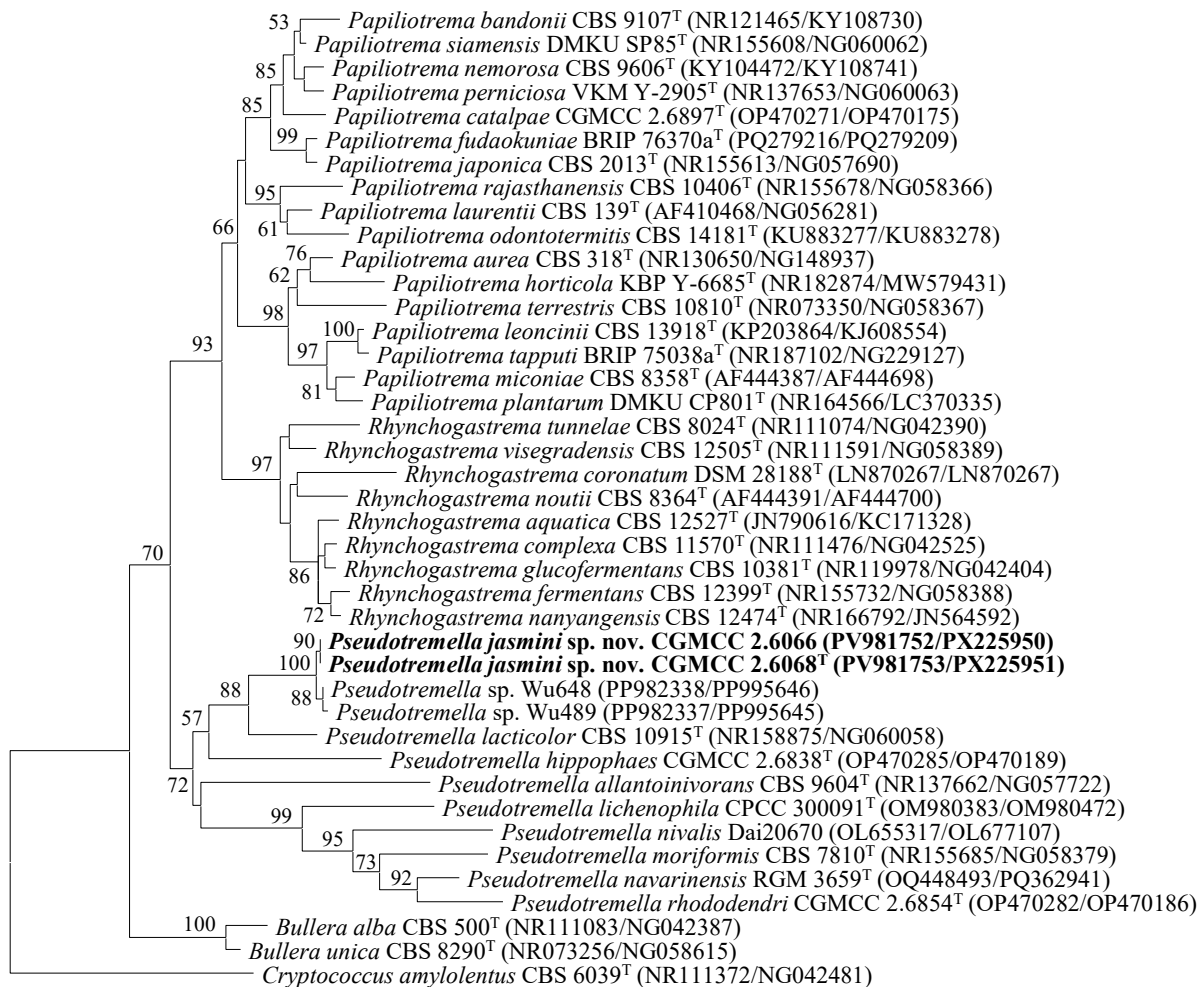

—————  
 0.02

Supplement: Supplementary file 1 [file jof-12-00521-s001.zip › Figure S2/Figure S2c-- 2.6068---NJ tree.pdf]

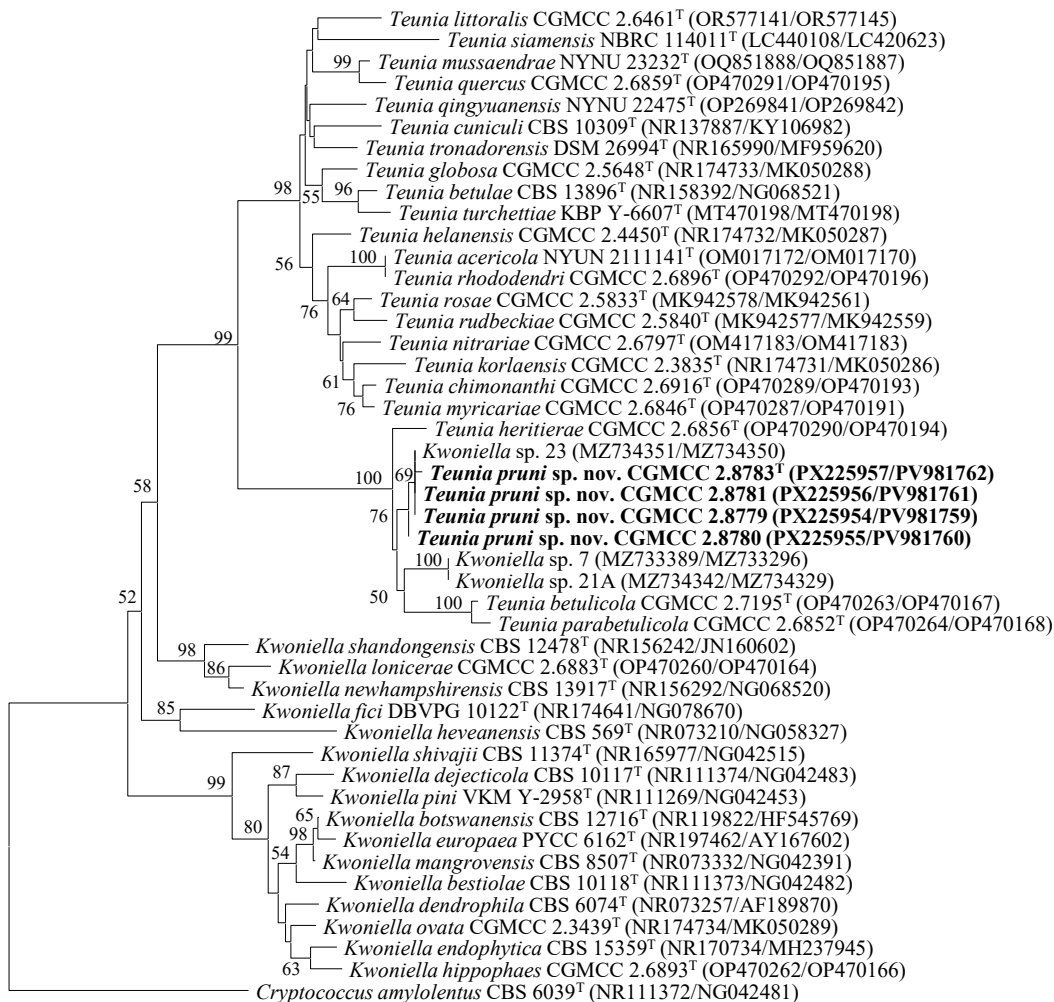

0.02

Supplement: Supplementary file 1 [file jof-12-00521-s001.zip › Figure S2/Figure S2d --2.8783---NJ tree.pdf]

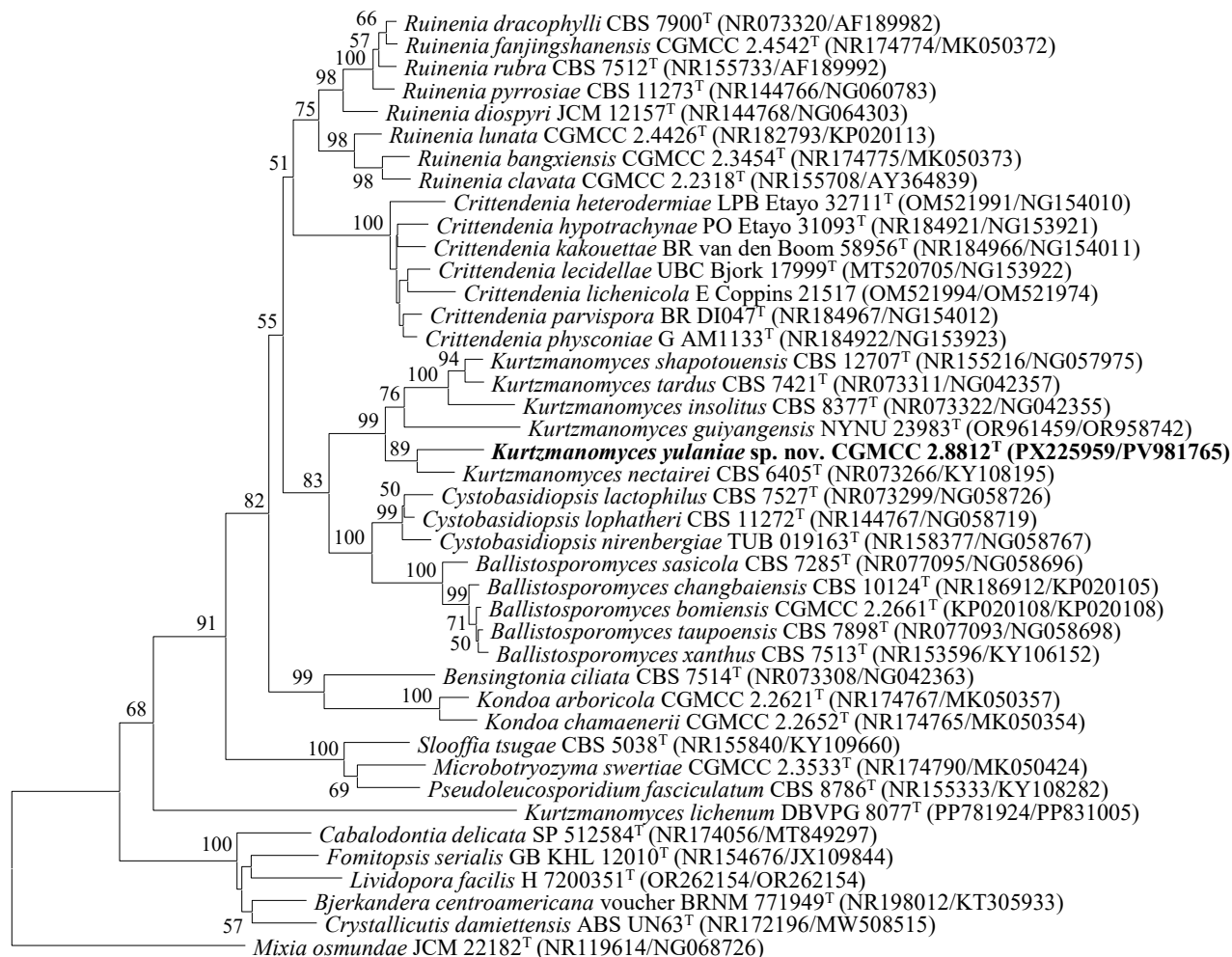

0.050

Supplement: Supplementary file 1 [file jof-12-00521-s001.zip › Figure S2/Figure S2e-- 2.8812---NJ tree.pdf]

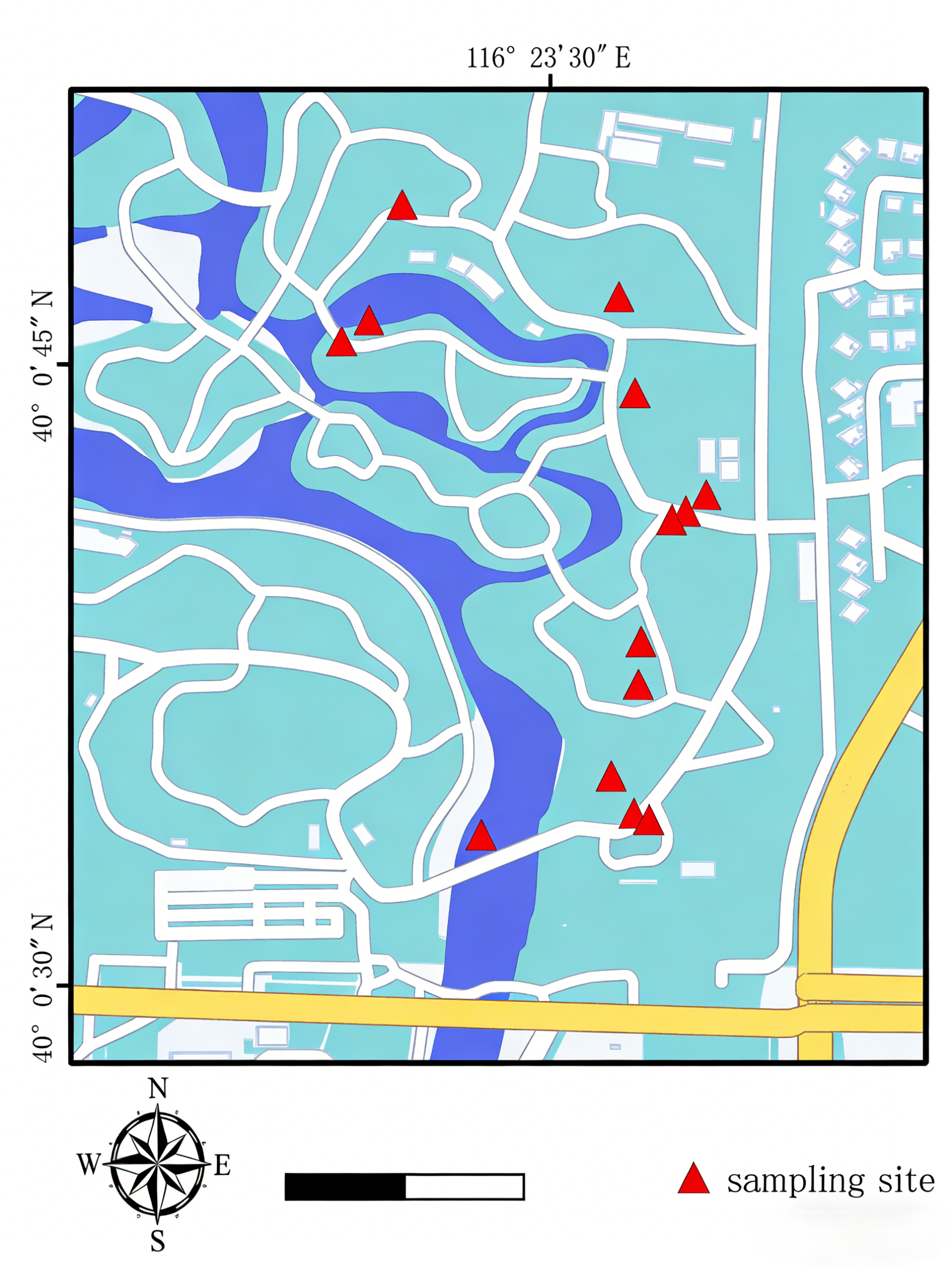

Supplement: Supplementary file 1 [file jof-12-00521-s001.zip › Figure S1.png]

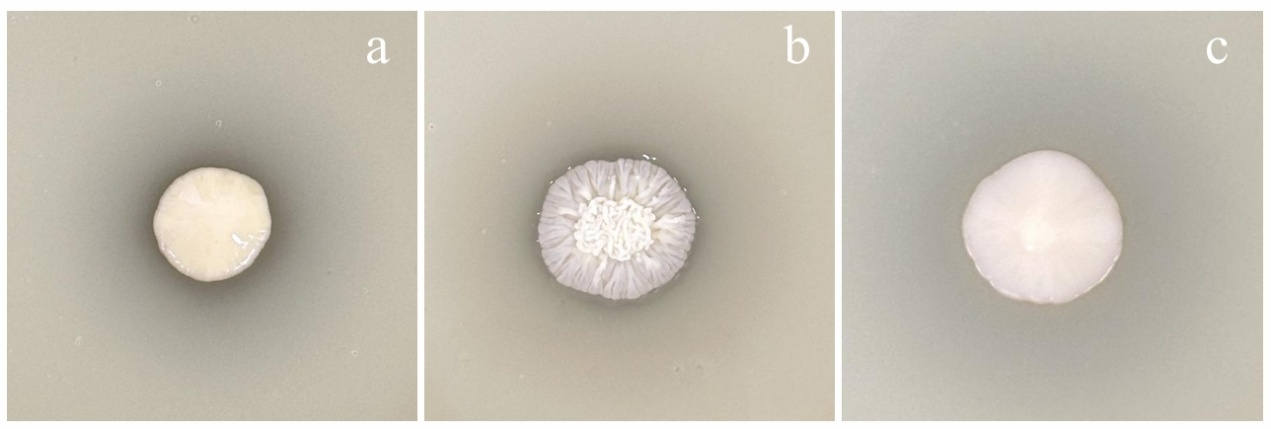

Supplement: Supplementary file 1 [file jof-12-00521-s001.zip › Figure S3.png]
